# Enrichment of microsomes from Chinese hamster ovary cells by subcellular fractionation for its use in proteomic analysis

**DOI:** 10.1371/journal.pone.0237930

**Published:** 2020-08-25

**Authors:** Saumel Pérez-Rodriguez, María de Jesús Ramírez-Lira, Tune Wulff, Bjørn Gunnar Voldbor, Octavio T. Ramírez, Mauricio A. Trujillo-Roldán, Norma A. Valdez-Cruz

**Affiliations:** 1 Programa de Investigación de Producción de Biomoléculas, Departamento de Biología Molecular y Biotecnología, Instituto de Investigaciones Biomédicas, Universidad Nacional Autónoma de México, Cd. Universitaria, Coyoacán, Ciudad de México, México; 2 The Novo Nordisk Foundation Center for Biosustainability, Technical University of Denmark, Kgs, Lyngby, Denmark; 3 Departamento de Medicina Molecular y Bioprocesos, Instituto de Biotecnología, Universidad Nacional Autónoma de México, Colonia Chamilpa, Cuernavaca, Morelos, México; University College Dublin, IRELAND

## Abstract

Chinese hamster ovary cells have been the workhorse for the production of recombinant proteins in mammalian cells. Since biochemical, cellular and omics studies are usually affected by the lack of suitable fractionation procedures to isolate compartments from these cells, differential and isopycnic centrifugation based techniques were characterized and developed specially for them. Enriched fractions in intact nuclei, mitochondria, peroxisomes, *cis*-Golgi, *trans*-Golgi and endoplasmic reticulum (ER) were obtained in differential centrifugation steps and subsequently separated in discontinuous sucrose gradients. Nuclei, mitochondria, *cis*-Golgi, peroxisomes and smooth ER fractions were obtained as defined bands in 30–60% gradients. Despite the low percentage represented by the microsomes of the total cell homogenate (1.7%), their separation in a novel sucrose gradient (10–60%) showed enough resolution and efficiency to quantitatively separate their components into enriched fractions in *trans*-Golgi, *cis*-Golgi and ER. The identity of these organelles belonging to the classical secretion pathway that came from 10–60% gradients was confirmed by proteomics. Data are available via ProteomeXchange with identifier PXD019778. Components from ER and plasma membrane were the most frequent contaminants in almost all obtained fractions. The improved sucrose gradient for microsomal samples proved being successful in obtaining enriched fractions of low abundance organelles, such as Golgi apparatus and ER components, for biochemical and molecular studies, and suitable for proteomic research, which makes it a useful tool for future studies of this and other mammalian cell lines.

## Introduction

Subcellular fractionation of mammalian cells has been applied for the study of morphology, composition, structure and interactions between organelles [1–3], cellular and molecular biology [4] and, more recently, the cell composition through omics approaches [5–7]. Some advantages of fractionation include the study of cellular processes *in vitro* [8–11], protein tracking [12], analysis of post-translational modifications (PTM) [13] and protein composition [6,14,15]. Although different protocols have been developed for subcellular fractionation [16–19], a universal one is not feasible because of the differences in the structure and interactions of organelles, and the cytoskeleton arrangement that have led to modifications for each particular tissue or cell line.

Suspension Chinese Hamster Ovary (CHO) cells are the most employed mammalian host for the production of recombinant glycoproteins; around 84% of approved antibodies were produced in these cells from 2015 to 2018 [20]. Given the high biopharmaceutical value of this cell line, the standardization and optimization of specific fractionation protocols are crucial to obtain a deeper knowledge that leads to the development of new sub-lines with improved capacities for recombinant protein production. However, few fractionation protocols have been reported for these cells in their suspension format. On the other hand, as overproducers of recombinant proteins, about 150 published papers have been reported to date that use fractionation protocols oriented only to the isolation of one or few organelles in an adherent phenotype [9,21–26]. These articles have used wild type and mutant CHO cells for the study of vesicular transport [9,21], lipid composition of plasma membrane (PM) [22], biogenesis of peroxisomes [25], and the subcellular distribution of nsL-TP protein [26]; and for the isolation of Golgi membranes, PM, endoplasmic reticulum (ER), nuclei, mitochondria and lysosomes [23,24].

The high cross-contamination of fractions enriched in PM, Golgi apparatus, ER, lysosomes [23] and peroxisomes [25] due to insufficient fractionation steps without applying any additional methodology, impedes the use of some of these protocols for applications such as proteomics. However, fractionation, in combination with isobaric and metabolic labeling and bioinformatics resources, allows a proteomic analysis and unambiguous assignment of cellular proteins to organellar compartments, even with the expected cross contamination [27–30]. In spite of the availability of protocols, these methodologies are technically challenging and for some compartments like the secretion pathway could show low separation between ER, Golgi apparatus and the ER-Golgi intermediate compartment (ERGIC).

Hence, the aim of the present study was to develop and characterize a protocol for subcellular fractionation of recombinant CHO cells grown in suspension, through differential and isopycnic centrifugation, to obtain enriched fractions of most organelles to study their biology. Since the classical secretion pathway can often become a bottleneck to increase expression of recombinant proteins in CHO cells [31–33], we focused on the separation of its components by isopycnic centrifugation. Enrichment and isolation of ER and Golgi apparatus were improved compared to a previous protocol [13], by the design of a novel discontinuous sucrose gradient, which could be extended to the separation of the components of microsomes from other mammalian cell lines. This gradient could also be used for the comparative proteomic study of the organelles of the classical secretion pathway under different experimental culture conditions or cell phenotypes.

## Materials and methods

### Cell line and culture conditions

CHO DP-12 clone #1933 ATCC® CRL-12444^TM^ [34] was cultured in CDM4CHO medium (Hyclone, UT, USA) supplemented with 6 mM stable glutamine (Biowest LLC, MO, USA), 0.002 mg/ml Humulin N (Eli Lilly, IN, USA) and 200 nM methotrexate (Pfizer, NY, USA), at 37ºC in a 5% CO_2_ atmosphere, in a humidified incubator. Cells were seeded in duplicate at 0.5 x 10^6^ cells/ml and a viability higher than 95%, in 35 ml medium in 250 ml Erlenmeyer flasks, at 60 rpm (Bellco Glass, NJ, USA). Cell concentration and viability were recorded every 24 h by cell counting in a Neubauer chamber, using the trypan blue dye exclusion method.

### Cell homogenization

The protocol for subcellular fractionation of CHO cells is available at protocols.io (dx.doi.org/10.17504/protocols.io.bf9sjr6e). Cells were centrifuged at 185 x g for 5 min and washed twice in a cold phosphate buffer (137 mM NaCl, 2.7 mM KCl, 8.1 mM Na_2_HPO_4_, 1.8 mM KH_2_PO_4_). Pellet was suspended at 6.6 x 10^7^ cell/ml, in HEPES buffer (1 mM EDTA, 10 mM HEPES, pH 7.4), and incubated for 30 min on ice. 1 mM PMSF and 10% (v/v) SigmaFast Protease Inhibitor Cocktail (Sigma-Aldrich, Merck KGaA, Darmstadt, Germany) were added to the suspension. Cells were broken up with 25 strokes in a Dounce homogenizer, after which sucrose was added at 0.25 M to restore osmolarity.

### Differential centrifugation

The homogenate was distributed in 1.5 ml tubes at 1 ml per tube, and pellets collected at 3,000 x g for 10 min, 9,000 x g for 15 min, and 100,000 x g for one hour, were named nuclear, mitochondrial and microsomal, respectively. The supernatant from the last centrifugation was named the cytosol [35,36]. Open-top thin-wall polypropylene tubes, 14 x 89 mm, were used for all ultracentrifugation steps at velocities ≥ 100,000 x g. Pellets were solubilized in isoelectric focusing buffer (IEF, 7 M urea, 2 M thiourea, 2% [w/v] CHAPS, 40 mM dithiothreitol) for characterization of differential centrifugation. Otherwise, pellets were stored on ice for their further separation in sucrose gradients.

### Isopycnic centrifugation

Nuclear, mitochondrial and microsomal pellets were diluted in 0.25 M sucrose, deposited on top of gradients and centrifuged at an average of 154,693 x g for 3 h (Optima XE ultracentrifuge, Beckman Coulter, IN, USA). A previous gradient [35] was adapted for nuclear and mitochondrial suspensions, composed of 1 ml of 60% (w/v) and 3 ml of each of the following solutions: 55, 40 and 30% (w/v) sucrose. Microsomal preparations were separated in two different gradients. The first one was adapted from literature [13] to 1 ml of each of the following solutions: 60, 45, 40, 35, 30, 25, 20 and 15% (w/v) sucrose. The second one, designed in house, was formed by 1 ml 60% (w/v) and 2.5 ml of each of the following solutions: 45, 35, 30 and 10% (w/v) sucrose. Visible bands or 500 μl fractions were collected for further characterization.

### Determination of protein and sucrose concentration

Protein concentration was determined by Bradford assay in 96-well microplates, using Dye Reagent Concentrate (Bio-Rad, CA, USA) according to the manufacturer’s recommendations. Bovine serum albumin (GE Healthcare Bio-Sciences, MA, USA) was used as standard, and samples in IEF buffer were diluted 5 times in water. Sucrose concentration was measured in a 0–32% Brix hand-refractometer.

### SDS-PAGE

Samples were mixed with Laemmli buffer [37], incubated at 95ºC for 5 min, centrifuged at 16,000 x g for 5 min and applied to 12% or 15% SDS-polyacrylamide gels. Samples previously diluted in IEF buffer were not heated. Page Ruler Pre-stained Protein Ladder (Thermo Fisher Scientific, MA, USA) was used as a molecular weight marker. Electrophoretic resolution was achieved in a SE260 Mini Vertical Protein Electrophoresis System (Hoefer Inc., MA, USA), using Tris-Glycine (25 mM Tris, 192 mM Glycine, 0.1% [w/v] SDS, pH 8.3) as running buffer. Gels were stained with Coomassie Brilliant Blue G 250 (Sigma-Aldrich, Merck KGaA, Darmstadt, Germany), and images acquired in a Gel DocTM EZ imager (Bio-Rad, CA, USA).

### Western blot (WB) identification

The protocol for detection of reference protein markers by WB, ELISA and enzymatic assays is available at protocols.io (dx.doi.org/10.17504/protocols.io.bgc4jsyw), and antibody information at S1 Table. The enrichment in ER, cytosol, nucleus, mitochondria, PM, *cis*-Golgi and *trans*-Golgi was assessed by detection of Grp78, Gapdh, histone H3, Hsp60, flotillin 1, golgin A5 and golgin-97 proteins, respectively. Membranes were revealed with SuperSignal West Pico Chemiluminescent Substrate kit (Thermo Fisher Scientific, MA, USA) and images acquired in a LI-COR C-DiGit Chemiluminescence Western Blot Scanner (LI-COR Biosciences, NE, USA).

Grp78, Gapdh and histone H3 were identified with 250X Endoplasmic Reticulum Fraction Western Blot Cocktail and 2500X horseradish peroxidase (HRP) Conjugated Secondary Antibody Cocktail (ab139415, Abcam, Cambridge, MA, USA), diluted 2000 and 2500 times, respectively.

For golgin-97, primary antibody (GTX114445, GeneTex, CA, USA) and an anti-rabbit IgG conjugated to HRP (ab205718, Abcam, Cambridge, MA, USA) were both diluted 2000 times. Anti flotillin 1 (GTX104769, GeneTex, CA, USA), anti Hsp60 (GTX110089, GeneTex, CA, USA) and anti golgin A5 (GTX104255, GeneTex, CA, USA) were diluted 2000, 10000 and 2000 times, respectively.

### Anti golgin-97 ELISA

Golgin-97 was also quantified by ELISA in samples from differential centrifugation using an optimized protocol. High binding plates (Greiner Bio-One GmbH, Austria) were coated with 4 μg of proteins from solubilized pellets, and *Escherichia coli* and CHO cell homogenates, in 0.05 M sodium carbonate-bicarbonate buffer, pH 9.6, for 16 h, at 4ºC. Wells were blocked with 1% (w/v) bovine serum albumin and 0.05% Tween-20 in phosphate buffer. The plate was then incubated with anti golgin-97 and anti-rabbit IgG conjugated to HRP, diluted 2000 and 1000 times, respectively. SigmaFast OPD substrate (Sigma-Aldrich, Merck KGaA, Darmstadt, Germany) was prepared according to manufacturer’s recommendations and incubated for 15 min. The reaction was stopped with 10% (v/v) HCl and absorbance recorded at 490 nm. *E*. *coli* and CHO cell lysates were chosen as negative and positive controls, respectively.

### Image processing and quantification

WB images were processed using an ImageJ v1.52a (National Institutes of Health, MD, USA). Enrichment after differential centrifugation, expressed as fold change, was the ratio of the areas of the protein marker between each fraction and homogenate.

For 15–60% gradients, the enrichment was calculated in two different ways. When all fractions were present in the same membrane, it was the ratio between the area of the protein marker and the total amount of proteins applied to the lane. When fractions were distributed among different membranes, the homogenate functioned as an internal control, and the ratio between the areas of the protein marker and homogenate was calculated. This ratio was multiplied by the average area of homogenates to obtain the normalized area of the protein marker. Finally, the enrichment was calculated as the ratio between the normalized area of the protein marker and the total concentration of proteins applied to the lane. For 10–60% and 30–60% gradients, enrichment was expressed as the ratio between the area of the protein marker and the total amount of proteins applied to the lane.

### Catalase assay

Enrichment of peroxisomes was assessed by a catalase assay adapted from Iwase, *et al*. [38]. Twenty microliters of samples were mixed with 30 μl of 50 mM phosphate buffer (21 mM KH_2_PO_4_, 29 mM K_2_HPO_4_, pH 7.0), and 50 μl of 1% (v/v) Triton X-100 and placed in a 12x75 mm glass test tube. Fifty microliters of 30% (v/v) hydrogen peroxide were added and mixed. Specific activity of catalase was measured as the ratio between foam height (mm), after 5 min incubation, and protein quantity added to the tube (mg).

### Transmission electron microscopy (TEM)

Precipitates were fixed in 2.5% (v/v) glutaraldehyde and post-fixed in 1% (w/v) osmium tetroxide. Sucrose was washed out by dilution in phosphate buffer followed by ultracentrifugation. Precipitates were dehydrated in ethanol solutions and incubated in pure acetonitrile. Samples were embedded in freshly prepared Epon resin [39], cut in a Reichert-Jung ultramicrotome and double contrasted. Images were acquired in a JEOL-1200 EX II transmission electron microscope.

### Proteomic characterization of microsomes from 10–60% sucrose gradients

Protein bands from 10–60% sucrose gradients were precipitated by acetone, according to previous guidelines [40]. Pellets were solubilized in guanidinium buffer (6M guanidinium hydrochloride, 5 mM Tris [2-carboxyethyl] phosphine, 10 mM chloracetamide, 100 mM Tris-HCl, pH 8.5), incubated at 99°C for 10 min and trypsinized overnight [41]. The reaction was stopped with 0.5% (v/v) trifluoroacetic acid and 20 μg of digested peptides were stage tipped according to Rappsilber, J. *et al*. [42]. A capillary-flow UltiMate 3000 RSLCnano system (capLC, Thermo Fisher Scientific, MA, USA) coupled to an 15 cm C18 easy spray column 50 μm x 150 mm, 2 μm Acclaim PepMap C18 column, was used at flow rates of 1.2 μl/min (Thermo Fisher Scientific, MA, USA), in a stepped gradient (3–45% acetonitrile). Samples were sprayed into a Q-Exactive HF-X mass spectrometer (Thermo Fisher Scientific, MA, USA) operated in Data dependent acquisition mode. A fullscan was collected at 60,000 resolution; AGC Target 3.0e6; maximum injection time 50 ms, followed by up to 12 MS2 scans at 15,000 resolution, maximum injection time 30 ms, HCD collision energy 28% and dynamic exclusion 25 sec. During MaxQuant analysis, carbamidomethyl (C) and oxidation of methionine residues were set as fixed and variable modifications, respectively; one missed cleavage was allowed and the false discovery rate was set to 1%. CHO proteome (UP000001075) was used as reference while including a list of known contaminants. Tolerance was set to 20 ppm. The mass spectrometry proteomics data have been deposited to the ProteomeXchange Consortium via the PRIDE [43] partner repository with the dataset identifier PXD019778.

Contaminants and groups identified in reverse database or by PTM were discarded. Proteins were aligned against *Mus musculus* proteome (UP000000589) [44] and classified by PANTHER v14.0 [45,46] and DAVID v6.8 resources [47,48]. Venn diagrams were created using VENNY v2.1 web tool [49].

### Statistical analysis

The Kruskal-Wallis test by ranks and Post-hoc Conover test with Bonferroni correction, in R language, were used to detect significant statistical differences in the distribution of reference proteins between subcellular fractions [50,51]. The significance level chosen corresponds to a p-value<0.05.

## Results

### Enrichment of nuclei, mitochondria, microsomes and cytosol by differential centrifugation

CHO cells, sampled during the exponential growth phase (S1 Fig), were mechanically disrupted and subcellular compartments isolated by differential centrifugation, from which nuclear, mitochondrial and microsomal pellets, and the cytosol, were collected. SDS-PAGE showed a different protein pattern between all the collected samples (S2 Fig), with a maximum amount of 15 kDa proteins in the nuclear pellet.

**Fig 1:**
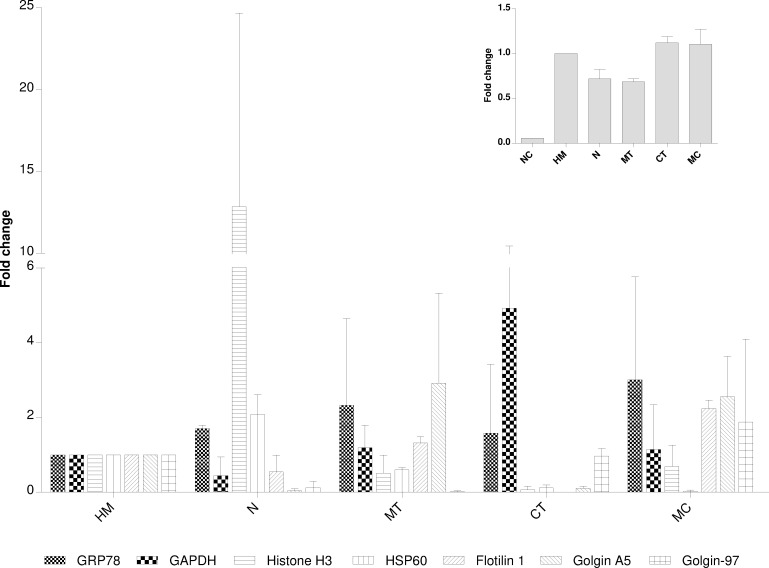
Western blot quantification of protein markers in differential centrifugation. Grp78, Gapdh, histone H3, Hsp60, flotillin 1, golgin A5 and golgin-97 were chosen as markers of endoplasmic reticulum, cytosol, nucleus, mitochondria, plasma membrane, *cis*-Golgi and *trans*-Golgi, respectively. Golgin-97 enrichment was confirmed by ELISA, as shown in the inset. HM: homogenate, N, MT, MC: nuclear, mitochondrial and microsomal precipitates, CT: cytosol. The *E*. *coli* homogenate was used as a negative control (NC) to verify any nonspecific recognition. Standard deviation was calculated from two biological replicates. No statistical differences were detected by Kruskal-Wallis test by ranks.

Enrichment of Grp78, histone H3, Gapdh, Hsp60, flotillin 1, golgin A5 and golgin-97, as markers of ER, nucleus, cytosol, mitochondria, PM, *trans*-Golgi and *cis*-Golgi, respectively, was verified by WB and ELISA (Fig 1 and S3 Fig), without statistical differences detected in these assays (p>0.05). The highest concentration of histone H3 and Gapdh was observed in the nuclear pellet and the cytosol, respectively. Hsp60 was mainly concentrated in nuclear and mitochondrial pellets, flotillin 1 and golgin A5 in mitochondrial and microsomal pellets, and golgin-97 in microsomal pellet and the cytosol. Grp78 was distributed among all pellets with a higher concentration in the microsomal one.

**Fig 2:**
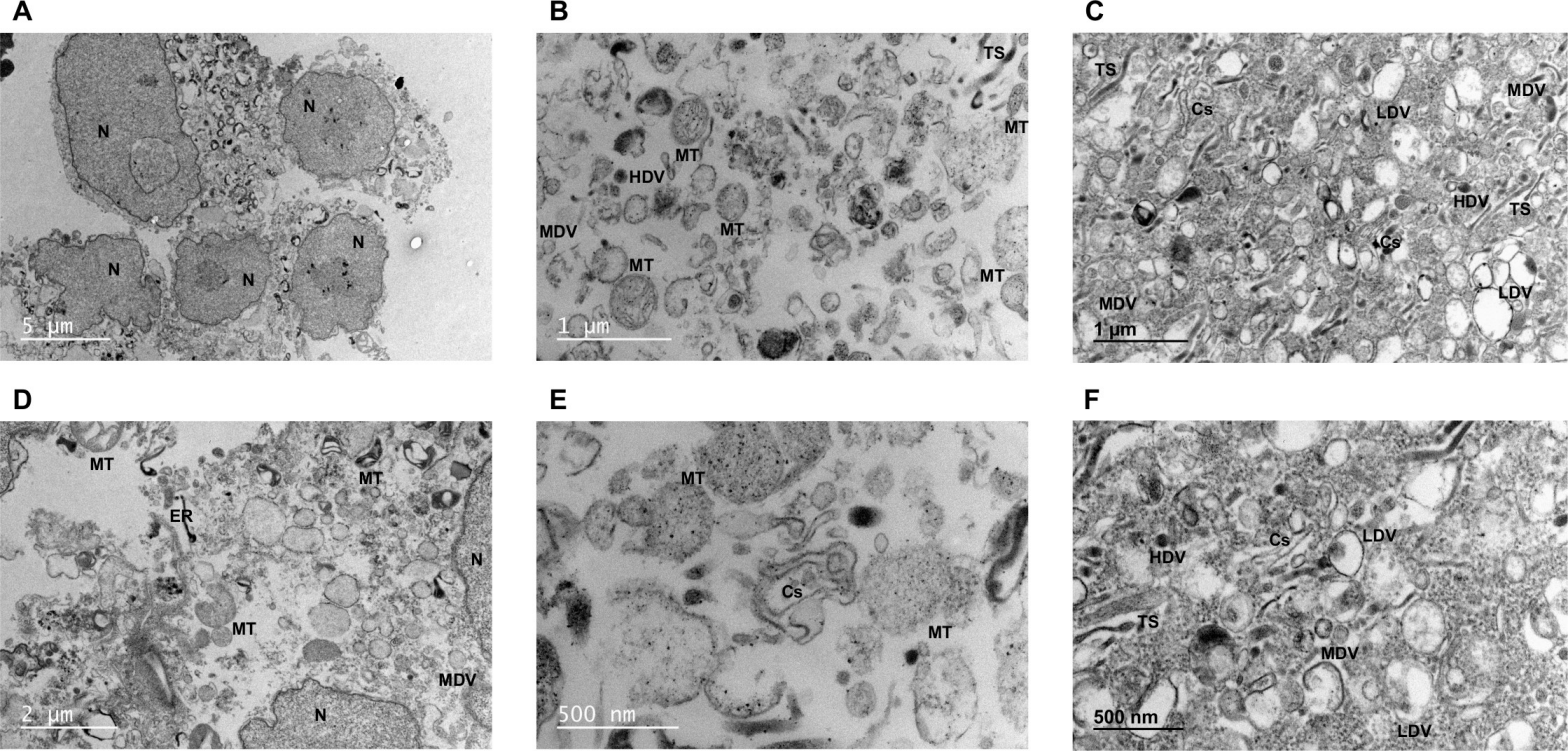
Electron microscopy characterization of samples from differential centrifugation. Images of nuclear (A, D), mitochondrial (B, E) and microsomal (C, F) pellets were acquired after fixation, staining and inclusion. According to their morphology, structures were classified as nucleus (N), mitochondria (MT), tubular structure (TS), cisternae (Cs), endoplasmic reticulum (ER) and low, medium and high-electrodensity vesicles (LDV, MDV and HDV, respectively). Two representative images were shown for each sample.

Morphological characterization showed that intact nuclei were enriched in the nuclear pellet (Fig 2A) together with mitochondria, ER and vesicles that remained entrapped in large structures around nuclei (Fig 2D). Abundant mitochondria, vesicles of different electrodensity, tubular structures (TS) and cisternae (Cs) were found in the mitochondrial pellet (Fig 2B–2E), while microsomes were only composed by TS, vesicles and Cs (Fig 2C–2F). Unexpectedly, Cs were more abundant in the mitochondrial than in the microsomal sample. Protein quantification revealed that almost all cellular protein content came from the nuclear pellet and cytosol, with a low representation of the mitochondrial and microsomal proteins (S2 Table).

### Separation and enrichment of organelles by isopycnic centrifugation

Differential fractionation is a fast, simple and effective technique for the isolation of crude fractions of cell organelles. However, a more efficient isolation and a higher degree of enrichment can be obtained from the separation of the differential centrifugation precipitates in density gradients. Therefore, nuclear and mitochondrial pellets were subsequently separated in 30–60% sucrose gradients, while for the microsomal precipitate, the performance of two different sucrose gradients (15–60% and 10–60%), was evaluated.

#### Enrichment of *trans*-Golgi, *cis*-Golgi and ER in 15–60% gradient

**Fig 3:**
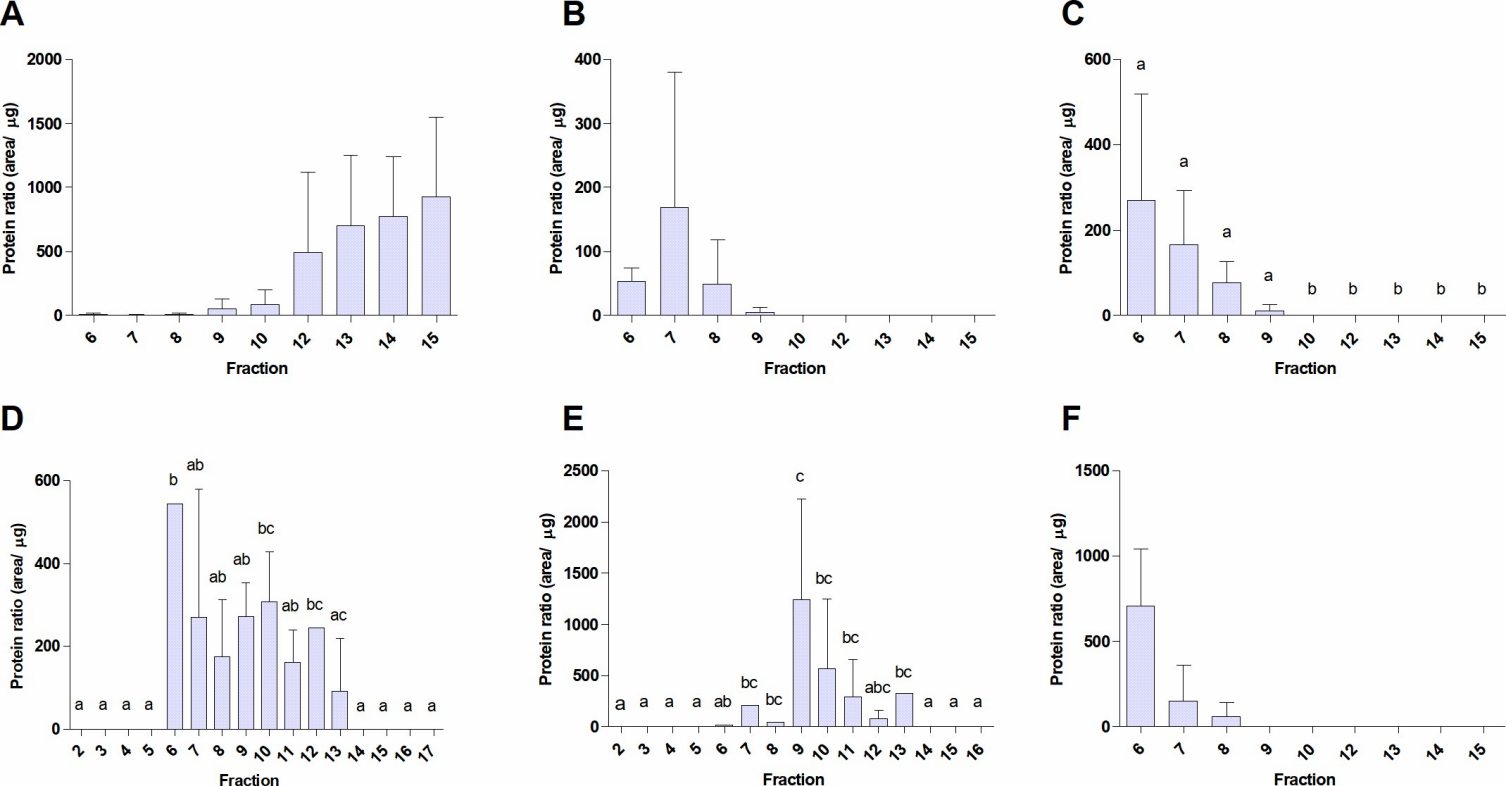
Western blot of microsomes in 15–60% gradients. Enrichment of Grp78 (A), histone H3 (C), Gapdh (B), flotillin 1 (D), golgin A5 (E) and golgin-97 (F) was quantified by Western blot in fractions collected and numbered from the top to the bottom of the tube (x-axis). Standard deviation was calculated from two biological replicates. Different letters indicate statistical differences (p≤0.05) according to the Kruskal-Wallis test by ranks and Post-hoc Conover test with Bonferroni correction, by contrast, fractions that share at least one letter are not significantly different from each other.

Separation and enrichment of microsomes were first evaluated in a 15–60% gradient, where three protein peaks (P1-P3) that corresponded to visible bands were observed (S4A and S4B Fig), with a partial overlapping between P1 and P2. Contamination with Gapdh (Fig 3B) and histone H3 (Fig 3C, p≤0.05) was present in lower density fractions (from 6 to 8), whereas flotillin 1 was mainly associated to medium density fractions (Fig 3D, p≤0.05). A large increase in intensity of golgin-97 in P1 (Fig 3F), golgin A5 in P2 (Fig 3E, p≤0.05) and Grp78 in P3 (Fig 3A) indicated that these fractions preferentially belonged to *trans*-Golgi, *cis*-Golgi and ER, respectively, as shown in Western blots (S5 Fig). Protein quantification, in agreement with SDS-PAGE (S4C and S4D Fig), showed that *cis*-Golgi (1.37 ± 0.01%) was more abundant than the *trans*-Golgi (0.59 ± 0.14%) and ER (0.85 ± 0.18%).

#### Enrichment of *trans*-Golgi, *cis*-Golgi and ER in 10–60% gradient

Given the low percentage represented by the microsomal proteins of the cell homogenate, it is relevant to maximize the separation and enrichment of the microsomal components, probably through an efficient sucrose gradient. This, coupled with the fact that a partial overlapping of Golgi domains in 15–60% gradient was observed, led to the design and characterization of a new 10–60% gradient in our laboratory.

**Fig 4:**
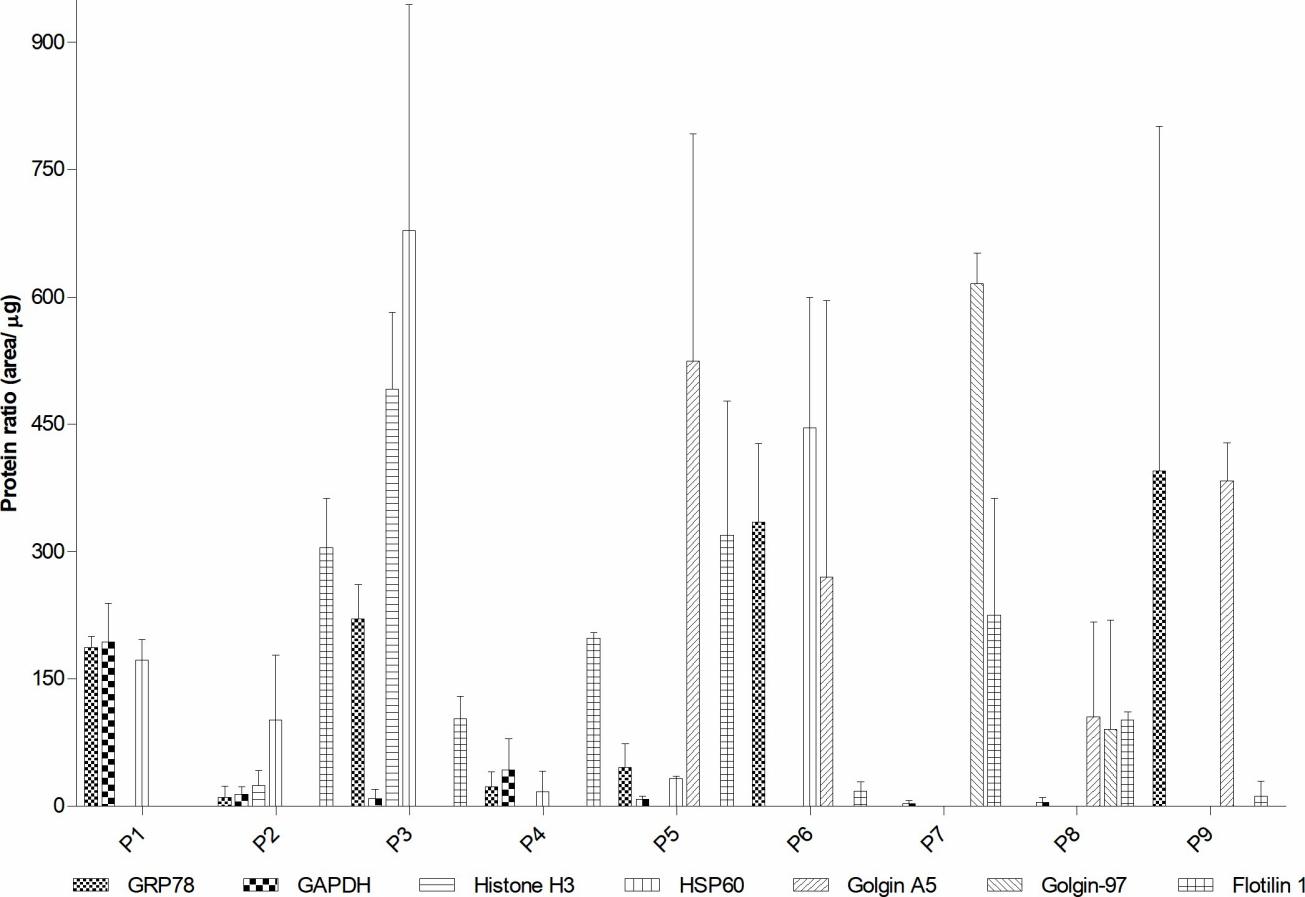
Western blot of 30–60% and 10–60% sucrose gradients. Protein peaks were collected in a 30–60% nuclear (P1-P3), 30–60% mitochondrial (P4-P6) and 10–60% microsomal (P7-P9) gradients and numbered from the top to the bottom of the tube. Enrichment of Grp78, histone H3, Gapdh, Hsp60, flotillin 1, golgin A5 and golgin-97 was quantified by Western blot. Standard deviation was calculated from two biological replicates.

After separation in this new gradient, three bands of greater visibility, definition and resolution were observed. These bands coincided with protein concentration peaks (P7-P9) and with the sharp increase in the concentration of sucrose (S6C Fig). Golgin-97 was mostly found in P7, a region of low-density microsomes, while golgin A5 migrated to medium and high-density zones (p≤0.05), and Grp78 was localized at peak P9, the highest density fraction. The main contamination, represented by flotillin 1, was observed fundamentally in medium to lower density areas (Fig 4 and S7 Fig, p≤0.05).

**Fig 5:**
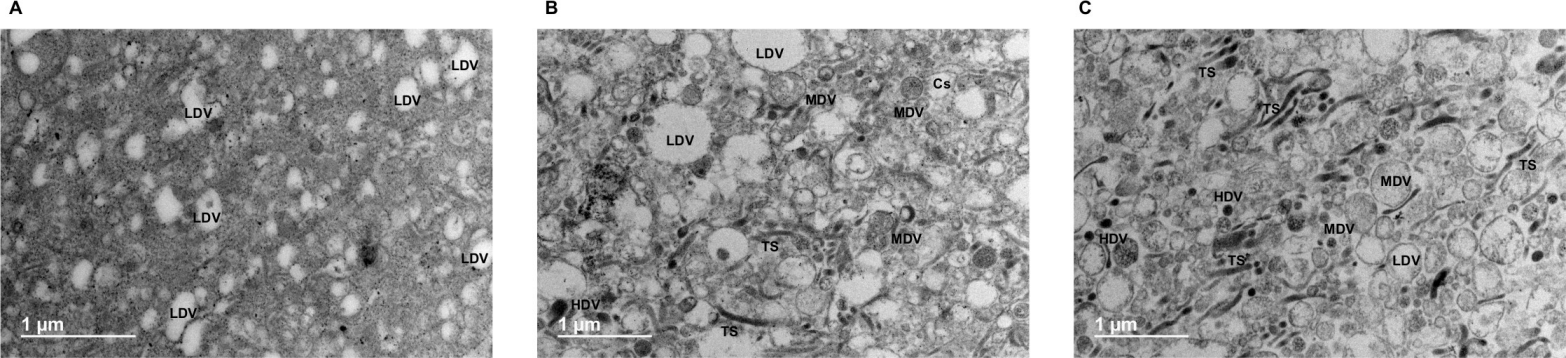
Electron microscopy characterization of microsomes in 10–60% sucrose gradients. According to their morphology, structures observed in P7 (A), P8 (B) and P9 (C) peaks, were classified as tubular structure (TS), cisternae (Cs) and low, medium and high-electrodensity vesicles (LDV, MDV and HDV, respectively). Representative images were shown for each sample.

P7 was composed almost exclusively of low-electrodensity vesicles (LDV) (Fig 5A), which together with medium-electrodensity vesicles (MDV), TS and Cs were also present in P8 (Fig 5B). The abundance of TS, MDV and high-electrodensity vesicles (HDV) increased in P9 (Fig 5C). These morphological characteristics, together with the WB results, showed an enrichment of *trans*-Golgi, *cis*-Golgi and ER in the P7-P9 fractions, respectively.

#### Separation of nuclear and mitochondrial components in 30–60% sucrose gradients

Besides the obtaining of fractions enriched in organelles of the classical secretion pathway, nuclear and mitochondrial precipitates were also separated and characterized, in order to provide a complete fractionation protocol. Similar to the microsomal separation, three visible and well resolved bands, coincident with protein peaks and sharp increases in sucrose concentration, were obtained in both gradients (S6A and S6B Fig), with a higher amount of proteins in the nuclear gradient (S3 Table). As a distinctive feature in SDS-PAGE (S6D Fig), strong protein bands between 10 and 15 kDa were only present in the highest density peak of the nuclear gradient (P3), which corresponded to histones (Fig 4 and S7 Fig, p≤0.05).

Grp78 was present in P1, P3 and P6 (Fig 4 and S7 Fig), coinciding with most of the catalase activity in P3 and P6 (S8 Fig). Hsp60 was located at the highest density peak of nuclear (P3) and mitochondrial (P6) gradients (p≤0.05). Flotillin 1 and golgin A5 showed the same migration pattern than in 10–60% microsomal gradients, while contamination with Gapdh was observed principally in the lower density region of the nuclear gradient (P1). This pattern of enrichment in nuclei, mitochondria, *cis*-Golgi and small vesicular organelles, detected by WB, was also confirmed by TEM (S9 Fig).

### Gene ontology classification of microsomal proteome from 10–60% sucrose gradient

**Fig 6:**
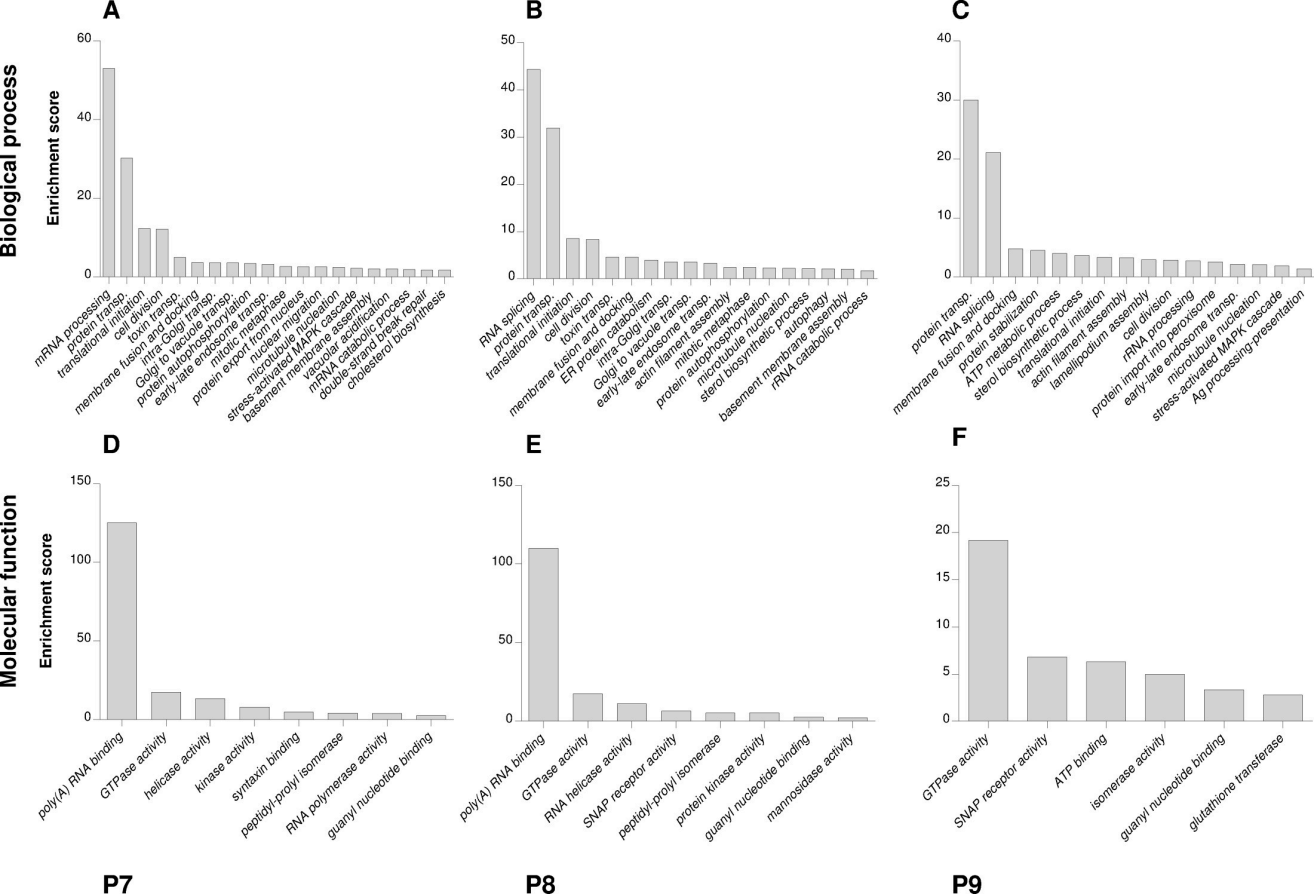
Classification of microsomal proteins by DAVID. Classification of proteins from P7 (A, D), P8 (B, E) and P9 (C, F) peaks, was done based on biological process (A-C) and molecular function (D-F), using DAVID functional annotation clustering. Transp.: transport.

Protein composition of microsomal bands collected from 10–60% sucrose gradients was elucidated by shot-gun proteomics (S1 File), and classified into Gene Ontology categories by DAVID and PANTHER algorithms. A total of 2913, 2737 and 1983 proteins were identified for P7-P9 peaks, respectively (S10 Fig). The three bands shared terms related to protein transport, vesicle docking and fusion, and protein folding in biological processes (Fig 6A–6C), molecular functions (Fig 6D–6F), functional classification (S11A–S11C Fig) and over-representation test (S12 Fig), confirming their belonging to organelles of the classical secretion pathway. P7 (Fig 6A) and P8 (Fig 6B) were enriched in Golgi transport terms, while mannosidase activity (Class II α-mannosidases), characteristic of *cis*-Golgi, was specific of P8 (Fig 6E). Protein catabolism and stress (S11C Fig), protein folding, protein targeting and localization to ER, and ER organization (S12C Fig) were some of the ER specific terms detected only in P9. Transition from Golgi apparatus (P7, P8) to ER (P9), was observed in cellular components (S11D–S11F and S12D–S12F Figs), biological processes (S12A–S12C Fig) and molecular functions (S12G–S12I Fig).

## Discussion

Subcellular fractionation has been widely employed for biochemical, physiological and cell biology research of tissues and cultured cells [3–5,8,11,13,52–55]. Since CHO cells are the workhorse for protein production in mammalian hosts, and a deeper knowledge of their biology could be obtained by fractionation, protocols to recover most of their organelles, based on differential and subsequent isopycnic centrifugation, were developed and characterized in this study. Within all organelles, those that belong to the classical secretion pathway play a preponderant role during the production of recombinant proteins, in many cases representing a bottleneck to increase productivity [31–33]. Thus, a greater emphasis was placed on microsome analysis, resulting in the design of a new sucrose gradient, fully compatible with proteomics, which will allow an accurate identification of the elements involved in increasing productivity.

Nuclear, mitochondrial and microsomal pellets, and the cytosol, were obtained from homogenates after differential centrifugation, where histone bands, of around 15 kDa in SDS-PAGE (S2 Fig), pointed to the preferential enrichment of nucleus in the nuclear pellet, a fact subsequently confirmed by WB (Fig 1) and TEM (Fig 2). The microsomal pellet was enriched in *trans*-Golgi (golgin-97), while *cis*-Golgi, tracked by golgin A5, was split into mitochondrial and microsomal pellets, as reported [56]. Cs were composed by denser structures in the mitochondrial pellet that precipitate before single tubules exhibited by the microsomal pellet, which could explain golgin A5 allocation. The wide distribution of PM (flotillin 1) could be associated with its very low density [7,14,19,22,56,57] and physical connections with ER [7,58], leading to its recognition as a common contaminant in centrifugation protocols [59]. ER (Grp78) was also broadly distributed among all subcellular compartments, being more concentrated in the microsomal pellet, in line with its distribution in other mammalian cells [7]. This wide presence of ER can be explained by physical proximity and in many cases the contacts with other organelles [3,58–60], with the exception of the cytosol, where its detection indicated some breakage of organelles by mechanical homogenization [24]. The location of mitochondria (Hsp60) in nuclear and mitochondrial precipitates was probably due to overlapped centrifugation velocities [19,36,59,61–63], different mitochondria populations [35,36,56], nuclei breakage and cytoskeleton aggregation [19,24,56,63,64].

Although it is good enough for nuclear and mitochondrial preparations [2,63,65,66], differential centrifugation is not suitable for obtaining individual fractions of other organelles such as ER, Golgi apparatus and endosomes [4,8,13,15,36]. Thus, since nuclear (unbroken cells, nuclei, mitochondria, ER, PM), mitochondrial (mitochondria, ER, PM, *cis*-Golgi) and microsomal pellets (PM, *trans*-Golgi, *cis*-Golgi, ER) were enriched in these specific organelles, a greater purity was then achieved in sucrose gradients, where nuclear, membrane and cytosolic proteins were detected as common contaminants [14,19,22,23,67].

Given the importance of the microsomes in protein expression, two different sucrose gradients were evaluated for a successful separation of this pellet. In 15–60% microsomal gradient, three visible bands were identified as *trans*-Golgi, *cis*-Golgi and ER, in increasing order of density, as has been confirmed for other biological samples [13,14,19,23,68]. However, as consequence of partial overlapping in this 15–60% gradient, a sharper 10–60% gradient was developed to increase the separation and yield of microsomes. Accordingly, three bands (S6C Fig), with an improved separation and focusing (S4A and S4B Fig) were obtained. The positions of *trans*-Golgi and ER remained the same as in the 15–60% gradient, while *cis*-Golgi was split between the two densest bands (Fig 4), probably caused by the migration of some domains of this organelle to a denser interface and co-localization with ER.

In 30–60% mitochondrial gradient, the highest density band was composed mainly by peroxisomes, *cis*-Golgi, ER and mitochondria, while of the studied markers, the medium density band only contained *cis*-Golgi. The distribution of *cis*-Golgi and mitochondria in fractions of different density in 30–60% gradients could be attributed to heterogeneous populations of these organelles [69–74] and their close contact with each other [75]. In the nuclear gradient, WB and TEM indicated the presence of smooth ER in the lowest density band, while the highest density band was substantially enriched in nuclear bodies. The final composition of compartments from isopycnic centrifugation is summarized in S13 Fig.

Given that previous protocols usually lack biological replicates [1,13,23,66], measurements of protein content [1,2,13,15,22,23,61,66] and standard deviation values [14], two advantages of the fractionation procedures characterized here are the quantitative distribution of protein markers [76], and the quantification of cellular proteins in each isolated fraction. More importantly, the new 10–60% sucrose gradient facilitates the isolation of fractions enriched in organelles from the classical secretion pathway, with greater separation, efficiency and resolution than the previous 15–60% gradient. Furthermore, it can be readily incorporated into common cell fractionation protocols during the separation of mammalian microsomes to study their composition, structure and functions.

To extend the use of the novel 10–60% gradient and characterize the protein composition of microsomal organelles, shot-gun proteomics was applied to fractions isolated from this gradient. As a result, proteome classification showed that all fractions maintained their expected functions in vesicle transport, folding and PTM of proteins. P7 and P8 fractions corresponded to Golgi apparatus according to Gene Ontology categories, and P8 band belonged specifically to *cis/medial*-Golgi due to its mannosidase II activity [77–79]. P9 represented the ER, because many of the identified biological processes, components and molecular functions only occur in this organelle [80–83]. Thus, besides being successful in obtaining enriched fractions of Golgi and ER components for biochemical and molecular studies, the new 10–60% sucrose gradient proved to be suitable for the proteomic characterization of these organelles.

## Conclusions

Subcellular fractionation has been widely employed for morphological, molecular, cellular and omics studies of tissues and cultured cells. Since CHO cells are the preferred host for expression of recombinant proteins in mammalian cells, differential and isopycnic centrifugation protocols were characterized and developed for this cell line. Given the essential role of organelles from the classical secretion pathway in protein expression, fractionation was focused mainly on the separation and enrichment of microsomal components. Crude preparations of nuclei, mitochondria, peroxisomes, microsomes and cytosol can be obtained by differential centrifugation, and subsequently enriched in sucrose gradients. Nuclei, *cis*-Golgi, smooth ER and mitochondria were isolated as individual bands in a 30–60% gradient, with peroxisomes enriched together with nuclei and mitochondria. The separation of *trans*-Golgi, *cis*-Golgi and ER organelles was improved in a new 10–60% sucrose gradient with respect to a previous 15–60% gradient, obtaining more visible and defined bands. The identity of these microsomal organelles was confirmed by proteomics, suggesting that this protocol is a useful tool for proteomic characterization of the classical secretion pathway, and for differential proteomic studies of these organelles between different cell phenotypes and cellular states. The usability, reproducibility and robustness of this new gradient indicate that it could also be extended to fractionation of other mammalian cells, for enrichment and study of the organelles from the classical secretion pathway. Molecular, biochemical and proteomic characterization of enriched organelles from CHO cells, obtained by the protocols described herein will help to understand their biology and to gain the necessary knowledge for the improvement of recombinant protein production.

## Supporting information

S1 Fig Growth kinetics of CRL-12444 cells. Cells were cultured in 250 ml Erlenmeyer flasks in CDM4CHO medium. Viable cell concentration (filled circles) and viability (empty circles) were determined by trypan blue dye exclusion method in a Neubauer chamber. Standard deviation was calculated from two biological replicates.
(EPS)

S2 Fig SDS-PAGE of samples from differential centrifugation. White asterisk indicated enrichment of proteins with a molecular weight of approximately 15 kDa in the nuclear precipitate. Black arrows indicated protein bands with differential distribution between subcellular compartments. Lanes: 1: Protein ladder, 2: Homogenate, 3–5: Nuclear, mitochondrial and microsomal precipitates, respectively, 6: Cytosol. Representative image of two biological replicates.
(PPTX)

S3 Fig Western blot of samples from differential centrifugation. Nuclear (N), mitochondrial (MT) and microsomal (MC) pellets, and cytosol (CT), were obtained from homogenates (HM) by differential centrifugation. Grp78 (A), Gapdh (A), histone H3 (A), Hsp60 (B), flotillin 1 (C), golgin A5 (D) and golgin-97 (E) were chosen as markers of endoplasmic reticulum, cytosol, nucleus, mitochondria, plasma membrane, *cis*-Golgi and *trans*-Golgi, respectively. Markers corresponding to predicted molecular weight are indicated by a black arrow, and its isoforms, when present, by an asterisk. Representative images of two biological replicates.
(PPTX)

S4 Fig Protein quantification and SDS-PAGE of microsomes in 15–60% gradients. Protein concentration (filled circles) and sucrose percentage (empty circles) were measured in fractions collected and numbered from the top to the bottom of the tube (x-axis, B). Band pattern was revealed by SDS-PAGE in reducing conditions (C, D). P1-P3: Peaks of protein concentration (A), PL: Protein ladder.
(PPTX)

S5 Fig Western blot of microsomal fractions in 15–60% sucrose gradient. Enrichment of Grp78 (A), Gapdh (A), histone H3 (A), flotillin 1 (B, C), golgin A5 (D, E) and golgin-97 (F, G) was verified by Western blot in fractions collected and numbered from the top to the bottom of the tube (2–17) corresponding to S4B Fig. Markers corresponding to predicted molecular weight are indicated by a black arrow, and its isoforms, when present, by an asterisk. Representative images of two biological replicates.
(PPTX)

S6 Fig Protein quantification and SDS-PAGE in 30–60% and 10–60% sucrose gradients. Nuclear (A) and mitochondrial (B) pellets were separated in a 30–60% gradient, and microsomal (C) pellet in a 10–60% gradient. Protein concentration (filled circles) and sucrose percentage (empty circles) were measured in protein peaks (P1-P9), collected and numbered from the top to the bottom of the tube (x-axis). Band pattern was revealed by SDS-PAGE in reducing conditions (D). White asterisks tagged intense bands of proteins between 10 and 15 kDa. PL: Protein ladder.
(PPTX)

S7 Fig Western blot of 30–60% and 10–60% sucrose gradients. Enrichment of Grp78 (A), Gapdh (A), histone H3 (A), Hsp60 (B), flotillin 1 (C), golgin A5 (D) and golgin-97 (E) was verified by Western blot in protein peaks from nuclear (P1-P3), mitochondrial (P4-P6) and microsomal (P7-P9) gradients, collected and numbered from the top to the bottom of the tube. Markers corresponding to predicted molecular weight are indicated by a black arrow, and their suggested isoforms, when present, by an asterisk. Representative images of two biological replicates.
(PPTX)

S8 Fig Catalase activity in 30–60% and 10–60% sucrose gradients. Specific activity was measured as the ratio between foam height (mm) and total amount of proteins (mg), in protein peaks collected in nuclear (P1-P3) and mitochondrial (P4-P6) gradients. No specific activity was detected in microsomal gradients (P7-P9). Standard deviation came from two biological replicates.
(EPS)

S9 Fig Electron microscopy characterization of 30–60% sucrose gradients. According to their morphology, structures observed in nuclear (A-C) and mitochondrial (D-F) gradients were classified as nucleus (N), mitochondria (MT), tubular structure (TS), cisternae (Cs), endoplasmic reticulum (ER) and low, medium and high-electrodensity vesicles (LDV, MDV and HDV, respectively). Peaks P1-P6 corresponded to A-F images, respectively. Representative images were shown for each sample.
(PPTX)

S10 Fig Microsomal proteome from P7-P9 protein peaks in 10–60% sucrose gradients. Peaks were collected, acetone precipitated and components identified by LC-MS/MS against Chinese hamster ovary reference proteome. Venn diagrams were drawn from identified proteins by VENNY v2.1 web tool.
(TIFF)

S11 Fig Classification of microsomal proteins by DAVID. Classification of proteins from P7 (A, D), P8 (B, E) and P9 (C, F) was done based on biological process (A-C) and cellular component (D-F) of DAVID functional annotation chart. Transp.: transport, FDR: False Discovery Rate.
(TIF)

S12 Fig Classification and mapping of microsomal proteins by PANTHER. Proteins from P7 (A, D, G), P8 (B, E, H) and P9 (C, F, I) were classified according to Gene Ontology terms (biological process [A-C], cellular component [D-F], and molecular function [G-I]) by using PANTHER overrepresentation test. Transp.: transport, assemb: assembly, act: activity.
(EPS)

S13 Fig Organelle composition of fractions from isopycnic centrifugation. Enrichment of subcellular compartments in visible bands/protein peaks from sucrose gradients was summarized. Assignment was made based on SDS-PAGE, Western blot, ELISA, catalase assay and transmission electron microscopy.
(PPTX)

S1 Table Antibody information. The recommended information of all the antibodies employed during this study is described.
(DOCX)

S2 Table Protein quantification in samples from differential centrifugation. Protein amount per million cells and its percentage was calculated for nuclear, mitochondrial and microsomal pellets, and cytosol, from differential centrifugation. Samples were solubilized in isoelectric focusing buffer and quantified by Bradford assay. The standard deviation came from two biological replicates.
(DOCX)

S3 Table Protein quantification of peaks from 30–60% and 10–60% sucrose gradients. Protein amount per million cells and its percentage were calculated for 9 protein peaks collected from isopycnic centrifugation in sucrose gradients (P1-P3: nuclear, P4-P6: mitochondrial, P7-P9: microsomal). Protein amount was quantified by the Bradford assay. The standard deviation came from two biological replicates.
(DOCX)

S1 File Proteomic characterization of microsomal fractions. Identification and quantification of microsomal proteins from P7 (samples 13–14), P8 (samples 15–16) and P9 (samples 17–18) fractions in 10–60% sucrose gradients by shot-gun proteomics, from two biological replicates. Protein groups were identified based on UP000001075 reference proteome. Data were depleted from contaminants and groups identified in reverse database or by PTM.
(XLSX)

S1 Raw images Raw immunoblot images were detected with SuperSignal West Pico chemiluminescent substrate kit from Thermo Fisher Scientific (34579), and images acquired in a LI-COR C-DiGit Chemiluminescence Western Blot Scanner (LI-COR Biosciences, NE, USA). Gels were stained with Coomassie Brilliant Blue G 250 from Sigma-Aldrich (B8647), and images acquired in a Gel DocTM EZ imager by using Image Lab software, v6.0.1 (Bio-Rad, CA, USA).
(PDF)
