# Supplementary figures and images for: Enrichment of microsomes from Chinese hamster ovary cells by subcellular fractionation for its use in proteomic analysis
Source: PLoS One. 2020 Aug 25;15(8):e0237930. doi: 10.1371/journal.pone.0237930 (PMC7447005; doi:10.1371/journal.pone.0237930)

## Slide 1
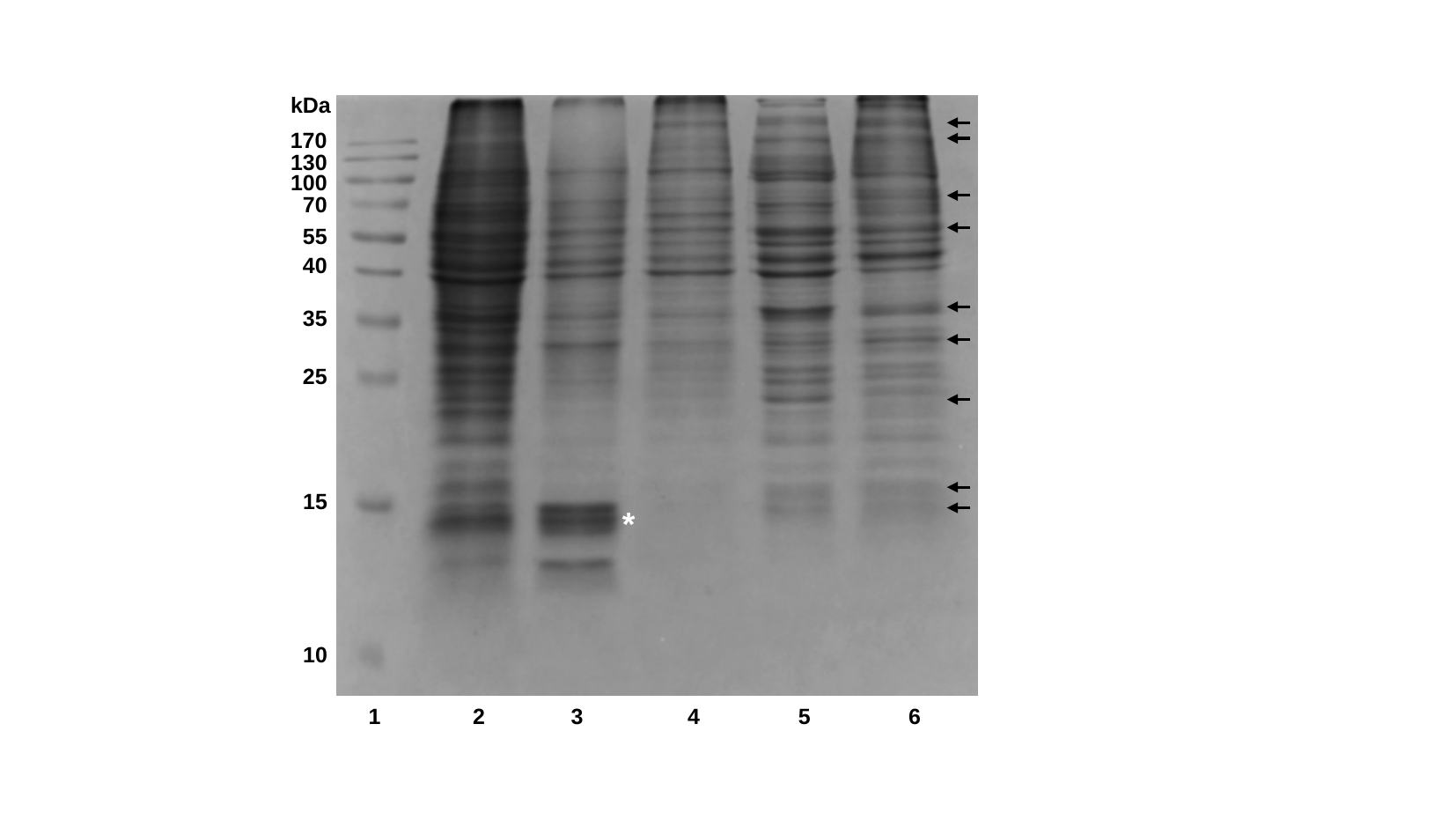

kDa
170
130
100
 70
 55
 40
 35
 25
 15
*
 10
 1 2 3 4 5 6

Supplement: S2 Fig — White asterisk indicated enrichment of proteins with a molecular weight of approximately 15 kDa in the nuclear precipitate. Black arrows indicated protein bands with differential distribution between subcellular compartments. Lanes: 1: Protein ladder, 2: Homogenate, 3–5: Nuclear, mitochondrial and microsomal precipitates, respectively, 6: Cytosol. Representative image of two biological replicates. (PPTX) [file pone.0237930.s002.pptx]

## Slide 1
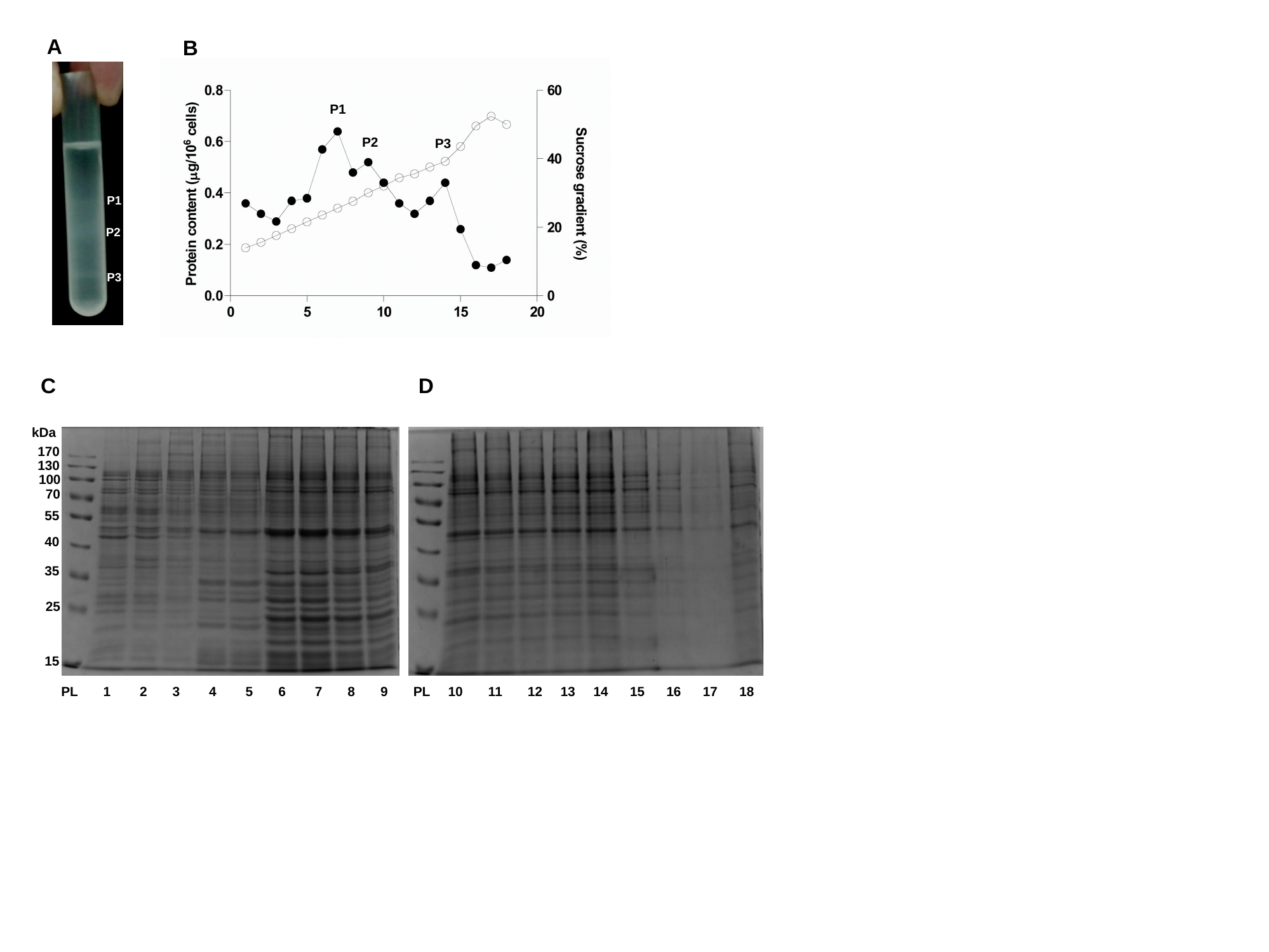

A
B
P1
P2
P3
P1
P2
P3
C
D
kDa
170
130
100
70
55
40
35
25
15
 PL 1 2 3 4 5 6 7 8 9 PL 10 11 12 13 14 15 16 17 18

Supplement: S4 Fig — Protein concentration (filled circles) and sucrose percentage (empty circles) were measured in fractions collected and numbered from the top to the bottom of the tube (x-axis, B). Band pattern was revealed by SDS-PAGE in reducing conditions (C, D). P1-P3: Peaks of protein concentration (A), PL: Protein ladder. (PPTX) [file pone.0237930.s004.pptx]

## Slide 1
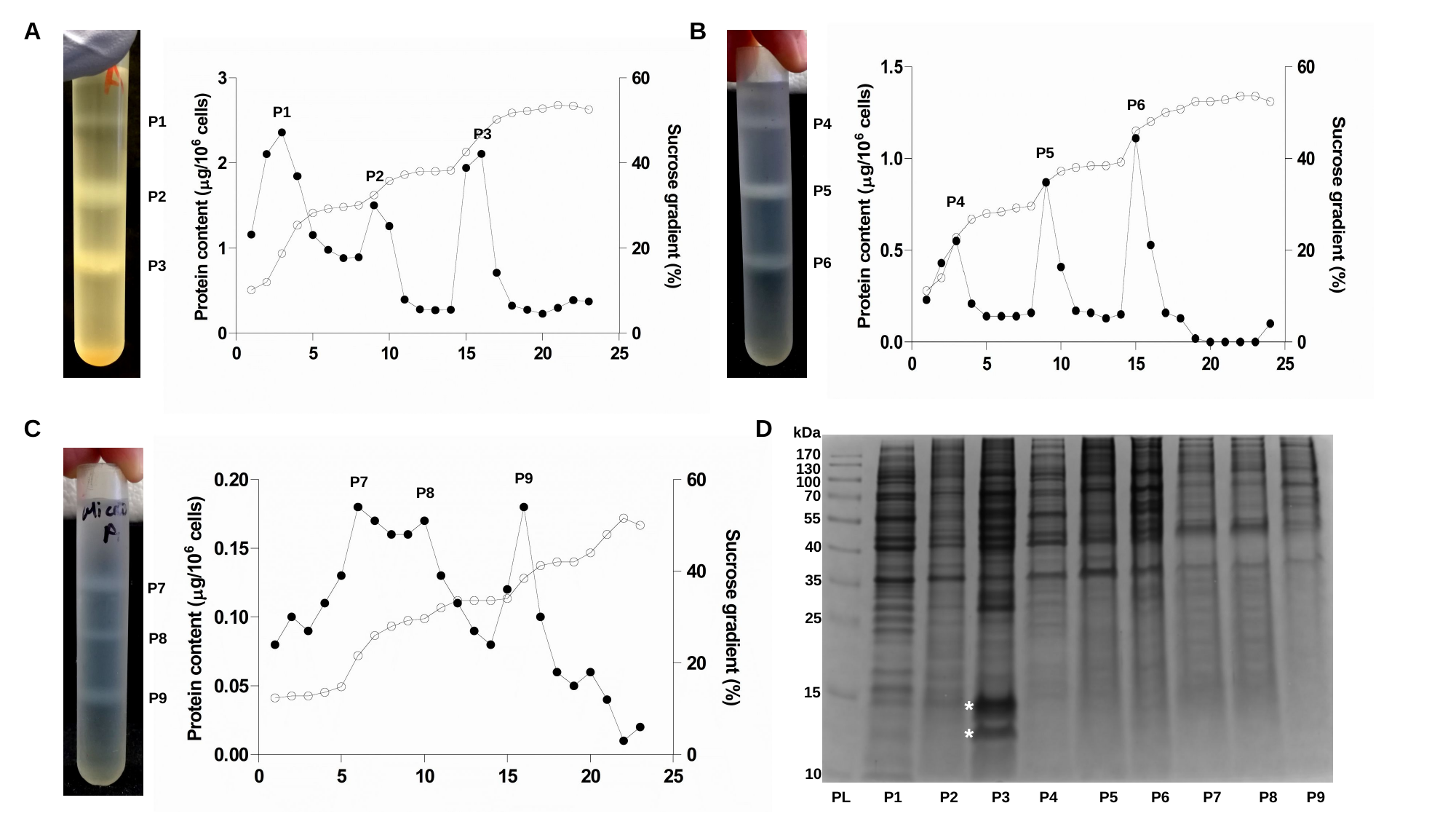

A
B
P6
P5
P4
P1
P2
P3
P4
P5
P6
P1
P3
P2
C
D
kDa
170
130
100
70
55
40
35
25
15
*
*
10
PL P1 P2 P3 P4 P5 P6 P7 P8 P9
P9
P7
P8
P7
P8
P9

Supplement: S6 Fig — Nuclear (A) and mitochondrial (B) pellets were separated in a 30–60% gradient, and microsomal (C) pellet in a 10–60% gradient. Protein concentration (filled circles) and sucrose percentage (empty circles) were measured in protein peaks (P1-P9), collected and numbered from the top to the bottom of the tube (x-axis). Band pattern was revealed by SDS-PAGE in reducing conditions (D). White asterisks tagged intense bands of proteins between 10 and 15 kDa. PL: Protein ladder. (PPTX) [file pone.0237930.s006.pptx]

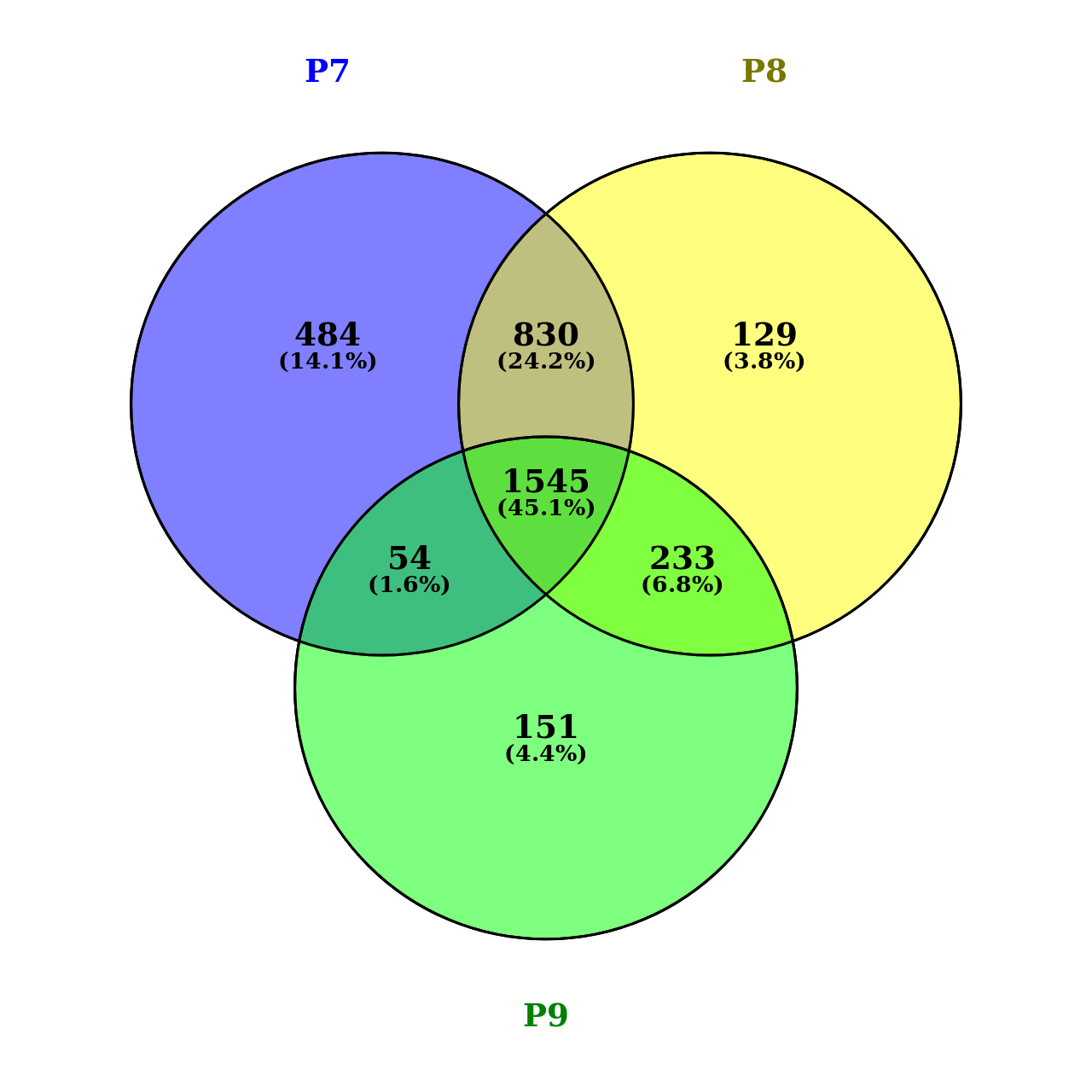

Supplement: S10 Fig — Peaks were collected, acetone precipitated and components identified by LC-MS/MS against Chinese hamster ovary reference proteome. Venn diagrams were drawn from identified proteins by VENNY v2.1 web tool. (TIFF) [file pone.0237930.s010.tiff]
