# Supplementary material for: Enrichment of microsomes from Chinese hamster ovary cells by subcellular fractionation for its use in proteomic analysis
Source: PLoS One. 2020 Aug 25;15(8):e0237930. doi: 10.1371/journal.pone.0237930 (PMC7447005; doi:10.1371/journal.pone.0237930)
Supplement: S3 Fig — Nuclear (N), mitochondrial (MT) and microsomal (MC) pellets, and cytosol (CT), were obtained from homogenates (HM) by differential centrifugation. Grp78 (A), Gapdh (A), histone H3 (A), Hsp60 (B), flotillin 1 (C), golgin A5 (D) and golgin-97 (E) were chosen as markers of endoplasmic reticulum, cytosol, nucleus, mitochondria, plasma membrane, cis-Golgi and trans-Golgi, respectively. Markers corresponding to predicted molecular weight are indicated by a black arrow, and its isoforms, when present, by an asterisk. Representative images of two biological replicates. (PPTX) [file pone.0237930.s003.pptx]

## Slide 1
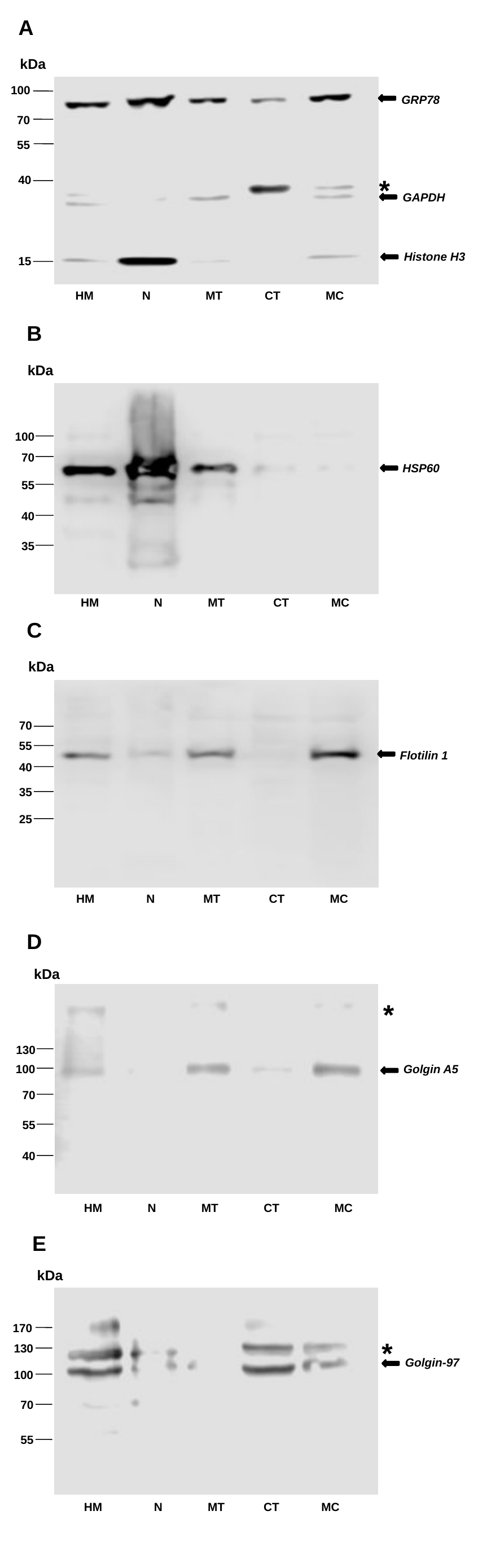

A
kDa
100
GRP78
70
55
*
40
GAPDH
Histone H3
15
 HM N MT CT MC
B
kDa
100
70
HSP60
55
40
35
 HM N MT CT MC
C
kDa
70
55
Flotilin 1
40
35
25
 HM N MT CT MC
D
kDa
*
130
100
Golgin A5
70
55
40
 HM N MT CT MC
E
kDa
170
*
130
Golgin-97
100
70
55
 HM N MT CT MC
