# Supplementary material for: Enrichment of microsomes from Chinese hamster ovary cells by subcellular fractionation for its use in proteomic analysis
Source: PLoS One. 2020 Aug 25;15(8):e0237930. doi: 10.1371/journal.pone.0237930 (PMC7447005; doi:10.1371/journal.pone.0237930)
Supplement: S5 Fig — Enrichment of Grp78 (A), Gapdh (A), histone H3 (A), flotillin 1 (B, C), golgin A5 (D, E) and golgin-97 (F, G) was verified by Western blot in fractions collected and numbered from the top to the bottom of the tube (2–17) corresponding to S4B Fig. Markers corresponding to predicted molecular weight are indicated by a black arrow, and its isoforms, when present, by an asterisk. Representative images of two biological replicates. (PPTX) [file pone.0237930.s005.pptx]

## Slide 1
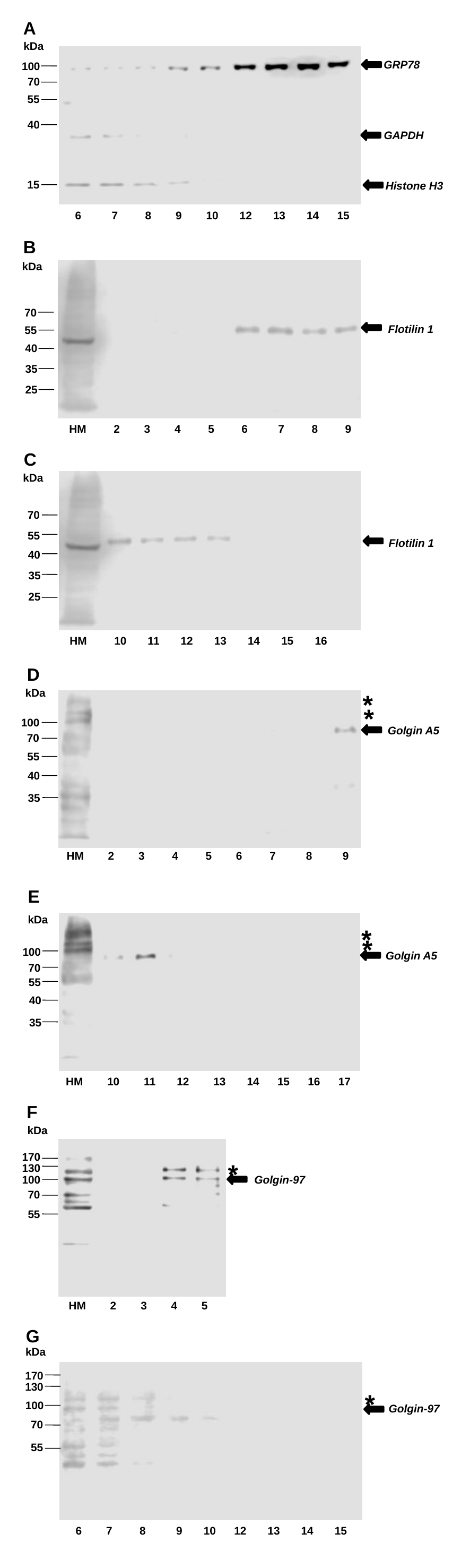

A
kDa
GRP78
100
70
55
40
GAPDH
15
Histone H3
 6 7 8 9 10 12 13 14 15
B
kDa
70
Flotilin 1
55
40
35
25
 HM 2 3 4 5 6 7 8 9
C
kDa
70
55
Flotilin 1
40
35
25
 HM 10 11 12 13 14 15 16
D
kDa
*
*
100
Golgin A5
70
55
40
35
HM 2 3 4 5 6 7 8 9
E
kDa
*
*
100
Golgin A5
70
55
40
35
HM 10 11 12 13 14 15 16 17
F
kDa
170
*
130
100
Golgin-97
70
55
 HM 2 3 4 5
G
kDa
170
130
*
100
Golgin-97
70
55
 6 7 8 9 10 12 13 14 15
