# Supplementary material for: Enrichment of microsomes from Chinese hamster ovary cells by subcellular fractionation for its use in proteomic analysis
Source: PLoS One. 2020 Aug 25;15(8):e0237930. doi: 10.1371/journal.pone.0237930 (PMC7447005; doi:10.1371/journal.pone.0237930)
Supplement: S7 Fig — Enrichment of Grp78 (A), Gapdh (A), histone H3 (A), Hsp60 (B), flotillin 1 (C), golgin A5 (D) and golgin-97 (E) was verified by Western blot in protein peaks from nuclear (P1-P3), mitochondrial (P4-P6) and microsomal (P7-P9) gradients, collected and numbered from the top to the bottom of the tube. Markers corresponding to predicted molecular weight are indicated by a black arrow, and their suggested isoforms, when present, by an asterisk. Representative images of two biological replicates. (PPTX) [file pone.0237930.s007.pptx]

## Slide 1
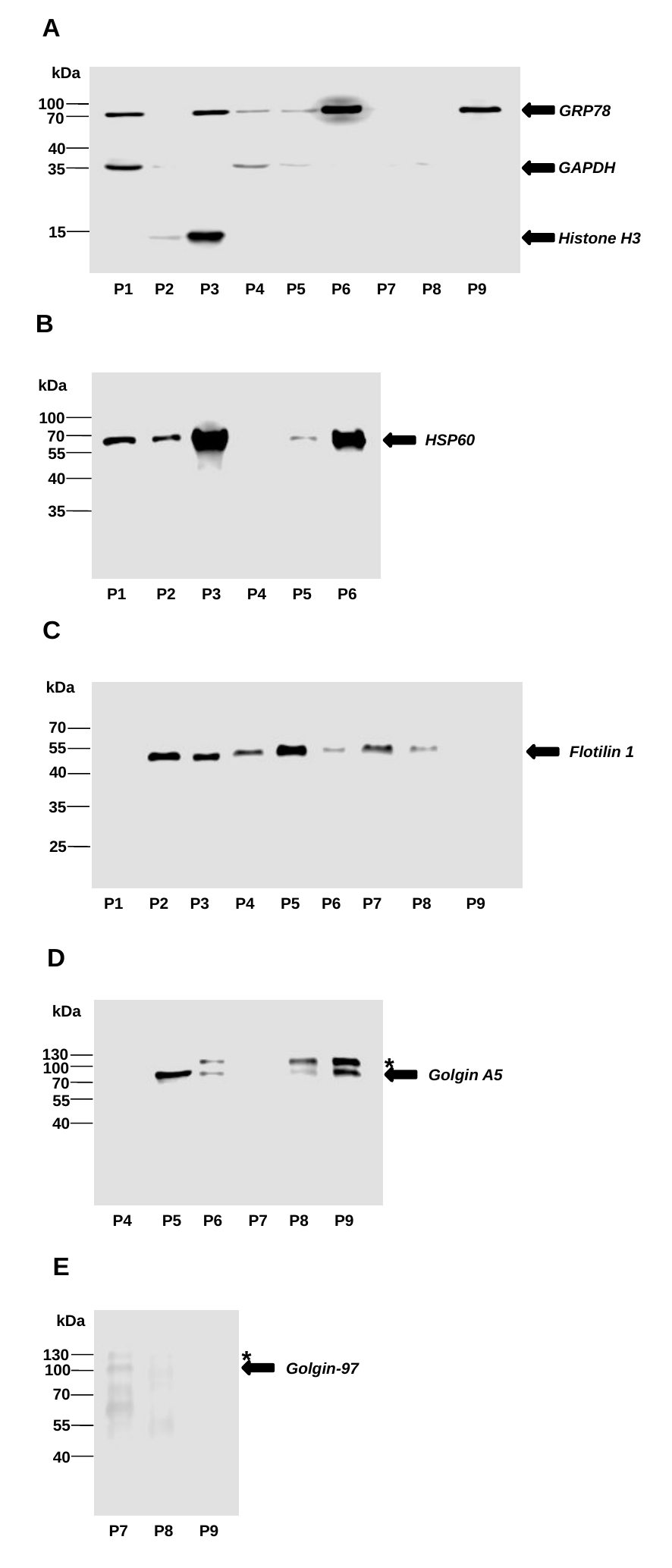

A
kDa
100
GRP78
70
40
GAPDH
35
15
Histone H3
 P1 P2 P3 P4 P5 P6 P7 P8 P9
B
kDa
100
70
HSP60
55
40
35
 P1 P2 P3 P4 P5 P6
C
kDa
70
55
Flotilin 1
40
35
25
 P1 P2 P3 P4 P5 P6 P7 P8 P9
D
kDa
130
*
100
Golgin A5
70
55
40
 P4 P5 P6 P7 P8 P9
E
kDa
*
130
Golgin-97
100
70
55
40
 P7 P8 P9
