# Supplementary material for: Enrichment of microsomes from Chinese hamster ovary cells by subcellular fractionation for its use in proteomic analysis
Source: PLoS One. 2020 Aug 25;15(8):e0237930. doi: 10.1371/journal.pone.0237930 (PMC7447005; doi:10.1371/journal.pone.0237930)
Supplement: S9 Fig — According to their morphology, structures observed in nuclear (A-C) and mitochondrial (D-F) gradients were classified as nucleus (N), mitochondria (MT), tubular structure (TS), cisternae (Cs), endoplasmic reticulum (ER) and low, medium and high-electrodensity vesicles (LDV, MDV and HDV, respectively). Peaks P1-P6 corresponded to A-F images, respectively. Representative images were shown for each sample. (PPTX) [file pone.0237930.s009.pptx]

## Slide 1
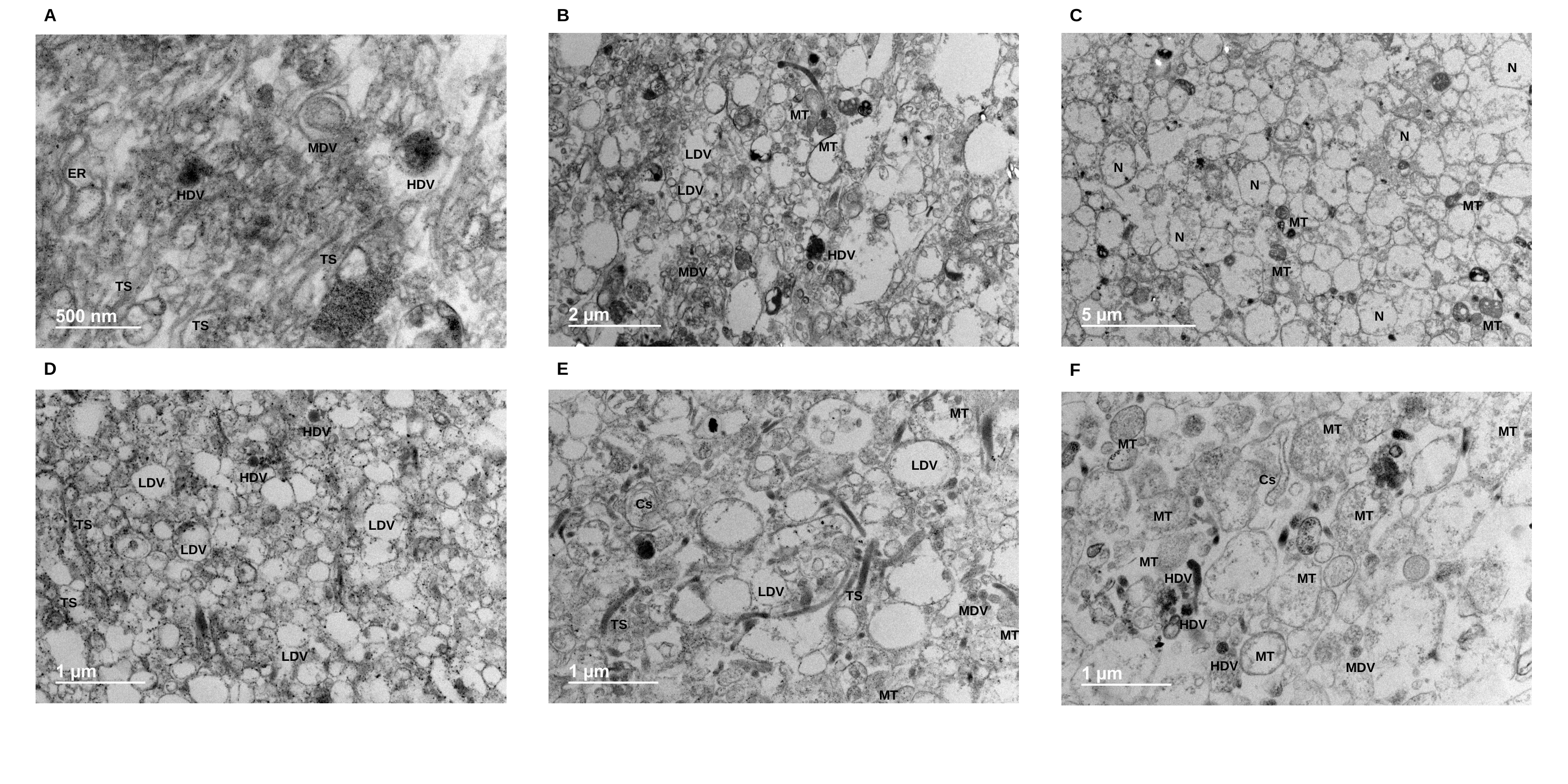

A
B
C
N
MT
N
MT
MDV
LDV
N
ER
HDV
N
LDV
HDV
MT
MT
N
HDV
TS
MT
MDV
TS
N
TS
MT
D
E
F
MT
MT
MT
HDV
MT
LDV
HDV
Cs
LDV
Cs
MT
MT
TS
LDV
LDV
MT
MT
HDV
LDV
TS
TS
MDV
HDV
TS
MT
LDV
MT
HDV
MDV
MT
