# Supplementary material for: Enrichment of microsomes from Chinese hamster ovary cells by subcellular fractionation for its use in proteomic analysis
Source: PLoS One. 2020 Aug 25;15(8):e0237930. doi: 10.1371/journal.pone.0237930 (PMC7447005; doi:10.1371/journal.pone.0237930)
Supplement: S13 Fig — Enrichment of subcellular compartments in visible bands/protein peaks from sucrose gradients was summarized. Assignment was made based on SDS-PAGE, Western blot, ELISA, catalase assay and transmission electron microscopy. (PPTX) [file pone.0237930.s013.pptx]

## Slide 1
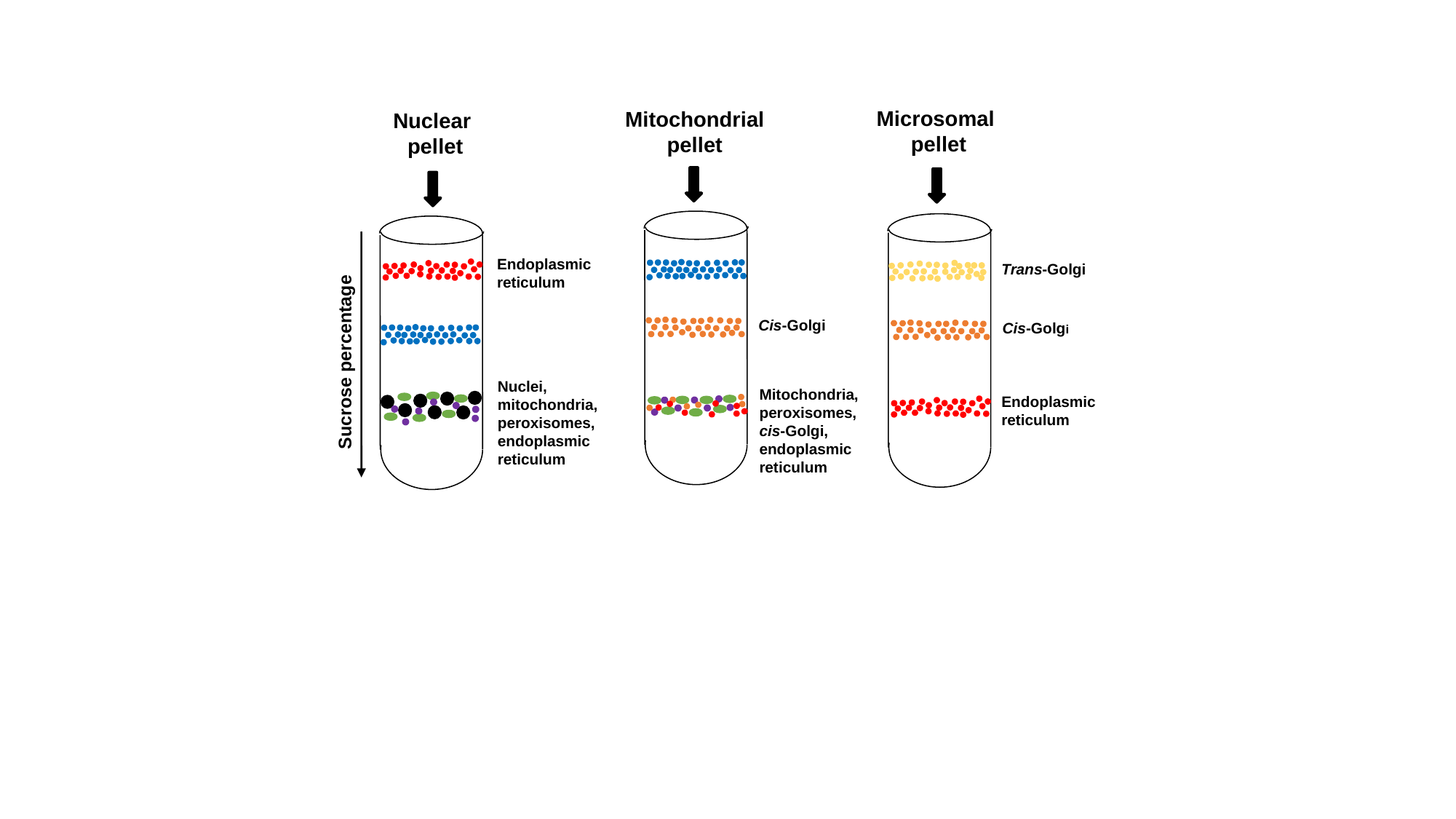

Microsomal
 pellet
Mitochondrial pellet
Nuclear
pellet
Endoplasmic reticulum
Trans-Golgi
Cis-Golgi
Cis-Golgi
Sucrose percentage
Nuclei, mitochondria, peroxisomes, endoplasmic reticulum
Mitochondria, peroxisomes, cis-Golgi, endoplasmic reticulum
Endoplasmic reticulum
