# Supplementary material for: Enrichment of microsomes from Chinese hamster ovary cells by subcellular fractionation for its use in proteomic analysis
Source: PLoS One. 2020 Aug 25;15(8):e0237930. doi: 10.1371/journal.pone.0237930 (PMC7447005; doi:10.1371/journal.pone.0237930)
Supplement: S1 Table — The recommended information of all the antibodies employed during this study is described. (DOCX) [file pone.0237930.s014.docx]

| **Name** | **Description** | **Supplier** | **Catalog number** | **Application/Dilution**^a^ | **Antigen** | **Identifier**^b^ |
| --- | --- | --- | --- | --- | --- | --- |
| 250X Endoplasmic Reticulum Fraction Western Blot Cocktail | 3 mAbs each targeting a specific organelle marker | Abcam, Cambridge, MA, USA | ab139415 | WB (1:2000) | GRP78  GAPDH  Histone H3 (di methyl K9) | AB_2847949 |
| 2500X HRP Conjugated Secondary Antibody Cocktail | Unknown | Abcam, Cambridge, MA, USA | ab139415 | WB (1:2500) | Unknown | AB_2847949 |
| Golgin 97 antibody [C2C3], C-term | Polyclonal rabbit IgG | GeneTex, CA, USA | GTX114445 | WB (1:2000)  ELISA (1:2000) | Recombinant protein encompassing a sequence within the C-terminus region of human Golgin 97. The exact sequence is proprietary. | AB_10619788 |
| Flotillin 1 antibody [C3], C-term | Polyclonal rabbit IgG | GeneTex, CA, USA | GTX104769 | WB (1:2000) | Carrier-protein conjugated synthetic peptide encompassing a sequence within the C-terminus region of human Flotillin 1. The exact sequence is proprietary. | AB_1240818 |
| HSP60 antibody | Polyclonal rabbit IgG | GeneTex, CA, USA | GTX110089 | WB (1:10 000) | Recombinant protein encompassing a sequence within the center region of human HSP60. The exact sequence is proprietary. | AB_1950529 |
| GOLGA5 antibody [N2C2], Internal | Polyclonal rabbit IgG | GeneTex, CA, USA | GTX104255 | WB (1:2000) | Recombinant protein encompassing a sequence within the center region of human GOLGA5. The exact sequence is proprietary. | AB_2037117 |
| Goat Anti-Rabbit IgG H&L (HRP) | Polyclonal goat IgG | Abcam, Cambridge, MA, USA | ab205718 | WB (1:2000)  ELISA (1:1000) | The details of the immunogen for this antibody are not available. | AB_2819160 |

^a^ WB stands for Western blot assay

^b^ A stable public identifier from the Antibody Registry (<https://antibodyregistry.org/>)
