# Supplementary material for: Enrichment of microsomes from Chinese hamster ovary cells by subcellular fractionation for its use in proteomic analysis
Source: PLoS One. 2020 Aug 25;15(8):e0237930. doi: 10.1371/journal.pone.0237930 (PMC7447005; doi:10.1371/journal.pone.0237930)
Supplement: S2 Table — Protein amount per million cells and its percentage was calculated for nuclear, mitochondrial and microsomal pellets, and cytosol, from differential centrifugation. Samples were solubilized in isoelectric focusing buffer and quantified by Bradford assay. The standard deviation came from two biological replicates. (DOCX) [file pone.0237930.s015.docx]

| **Sample** | **µg x 10^6^ cells** | **Percentage^a^** |
| --- | --- | --- |
| Homogenate | 201.24 ± 26.05 | 100.00 ± 0.00 |
| Nuclear | 96.21 ± 15.46 | 47.71 ± 1.51 |
| Mitochondrial | 10.87 ± 1.95 | 5.39 ± 0.27 |
| Cytosol | 90.62 ± 7.51 | 45.17 ± 2.11 |
| Microsomal | 3.54 ± 1.11 | 1.74 ± 0.32 |

^a^ Average value of both homogenates was set as 100 percent, and all further percentages were calculated in relation to that.
