# Supplementary material for: Enrichment of microsomes from Chinese hamster ovary cells by subcellular fractionation for its use in proteomic analysis
Source: PLoS One. 2020 Aug 25;15(8):e0237930. doi: 10.1371/journal.pone.0237930 (PMC7447005; doi:10.1371/journal.pone.0237930)
Supplement: S3 Table — Protein amount per million cells and its percentage were calculated for 9 protein peaks collected from isopycnic centrifugation in sucrose gradients (P1-P3: nuclear, P4-P6: mitochondrial, P7-P9: microsomal). Protein amount was quantified by the Bradford assay. The standard deviation came from two biological replicates. (DOCX) [file pone.0237930.s016.docx]

| **Sample** | **µg x 10^6^ cells** | **Percentage^a^** |
| --- | --- | --- |
| Homogenate | 84.79 ± 9.22 | 100.00 ± 0.00 |
| Cytosol | 43.62 ± 0.79 | 51.80 ± 6.56 |
| Nuclear gradient |  |  |
| P1^b^ | 8.16 ± 2.10 | 9.82 ± 3.55 |
| P2 | 4.68 ± 0.65 | 5.60 ± 1.37 |
| P3 | 6.77 ± 1.88 | 8.15 ± 3.10 |
| Mitochondrial gradient |  |  |
| P4 | 1.30 ± 0.26 | 1.52 ± 0.14 |
| P5 | 1.38 ± 0.43 | 1.61 ± 0.34 |
| P6 | 1.52 ± 0.73 | 1.76 ± 0.67 |
| Microsomal gradient |  |  |
| P7 | 0.62 ± 0.07 | 0.73 ± 0.00 |
| P8 | 0.62 ± 0.06 | 0.74 ± 0.15 |
| P9 | 0.48 ± 0.11 | 0.56 ± 0.07 |

^a^ Average value of both homogenates was set as 100 percent, and all further percentages were calculated in relation to that.

^b^ P1-P9 stands for protein peaks 1-9 collected from three sucrose gradients.
