# Supplementary material for: Enrichment of microsomes from Chinese hamster ovary cells by subcellular fractionation for its use in proteomic analysis
Source: PLoS One. 2020 Aug 25;15(8):e0237930. doi: 10.1371/journal.pone.0237930 (PMC7447005; doi:10.1371/journal.pone.0237930)

S2 Fig. Lanes: 1: protein ladder, 2: homogenate, 3-5: Nuclear, mitochondrial and microsomal precipitates respectively, 6: Cytosol.

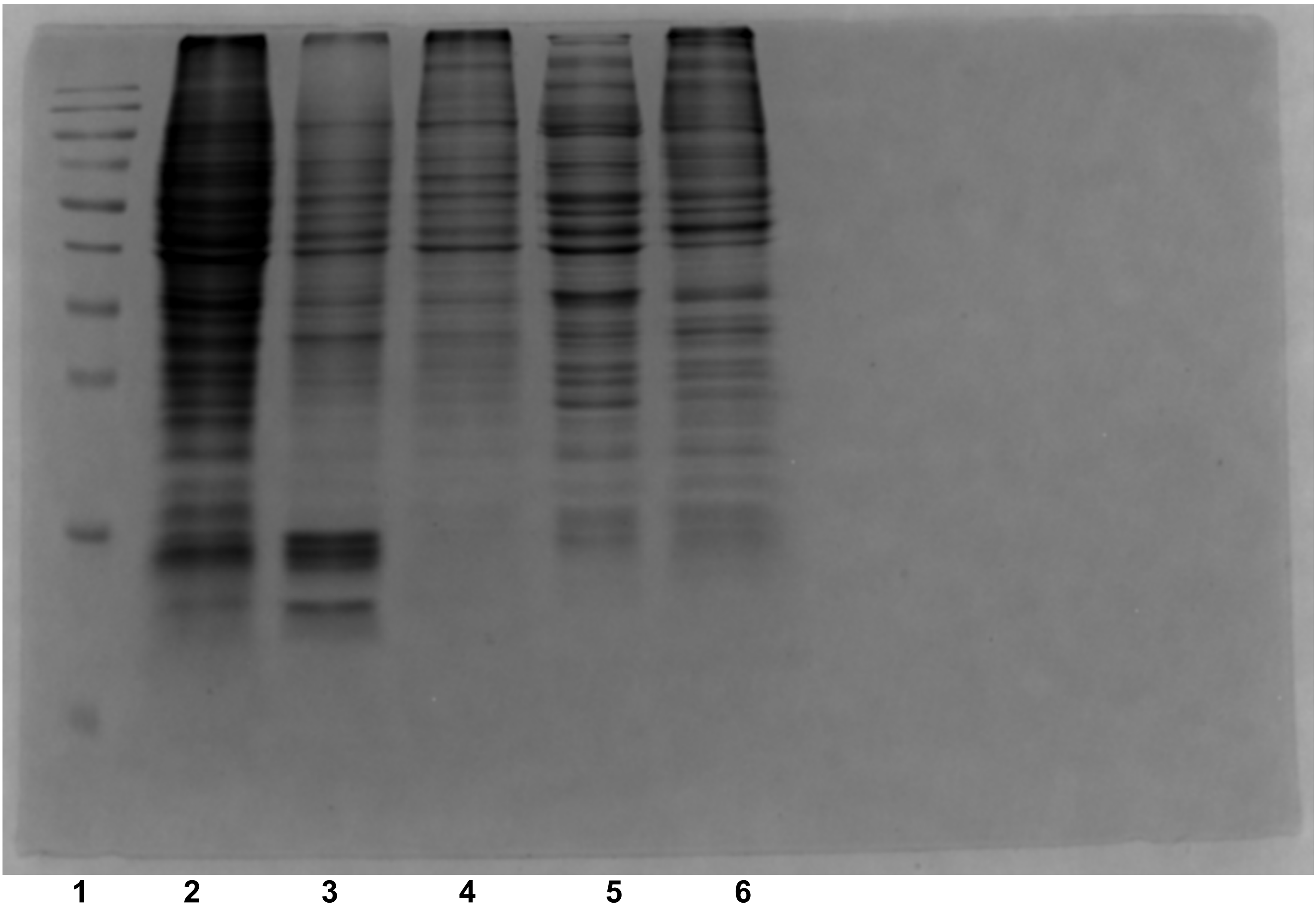

S3A Fig. Lanes: FL: plastic fluorescent marker, PL: protein ladder, HM: homogenate, N: Nuclear, MT: mitochondrial and MC: microsomal pellets, CT: cytosol.

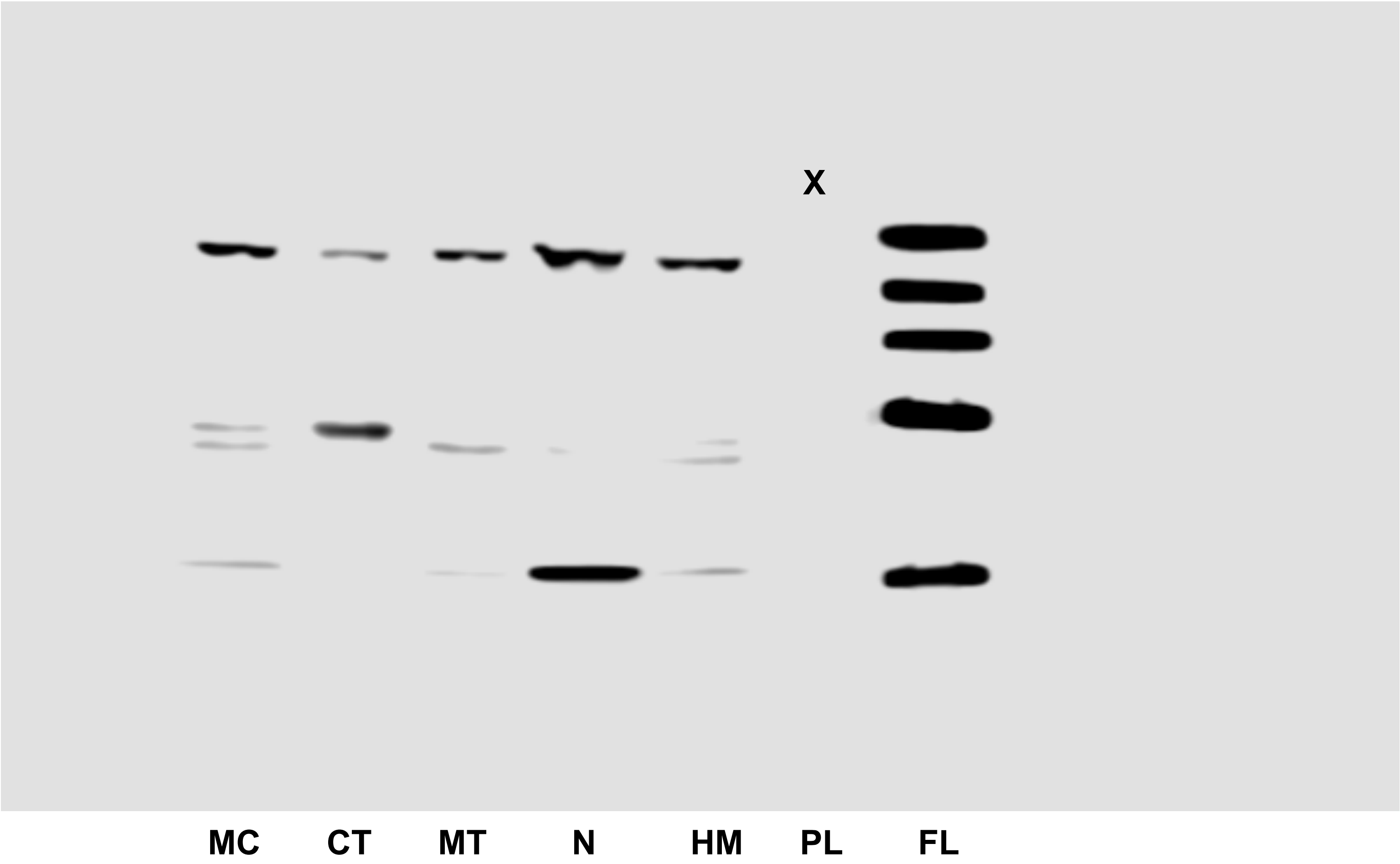

S3B Fig. Lanes: FL: plastic fluorescent marker, PL: protein ladder, HM: homogenate, N: Nuclear, MT: mitochondrial and MC: microsomal pellets, CT: cytosol.

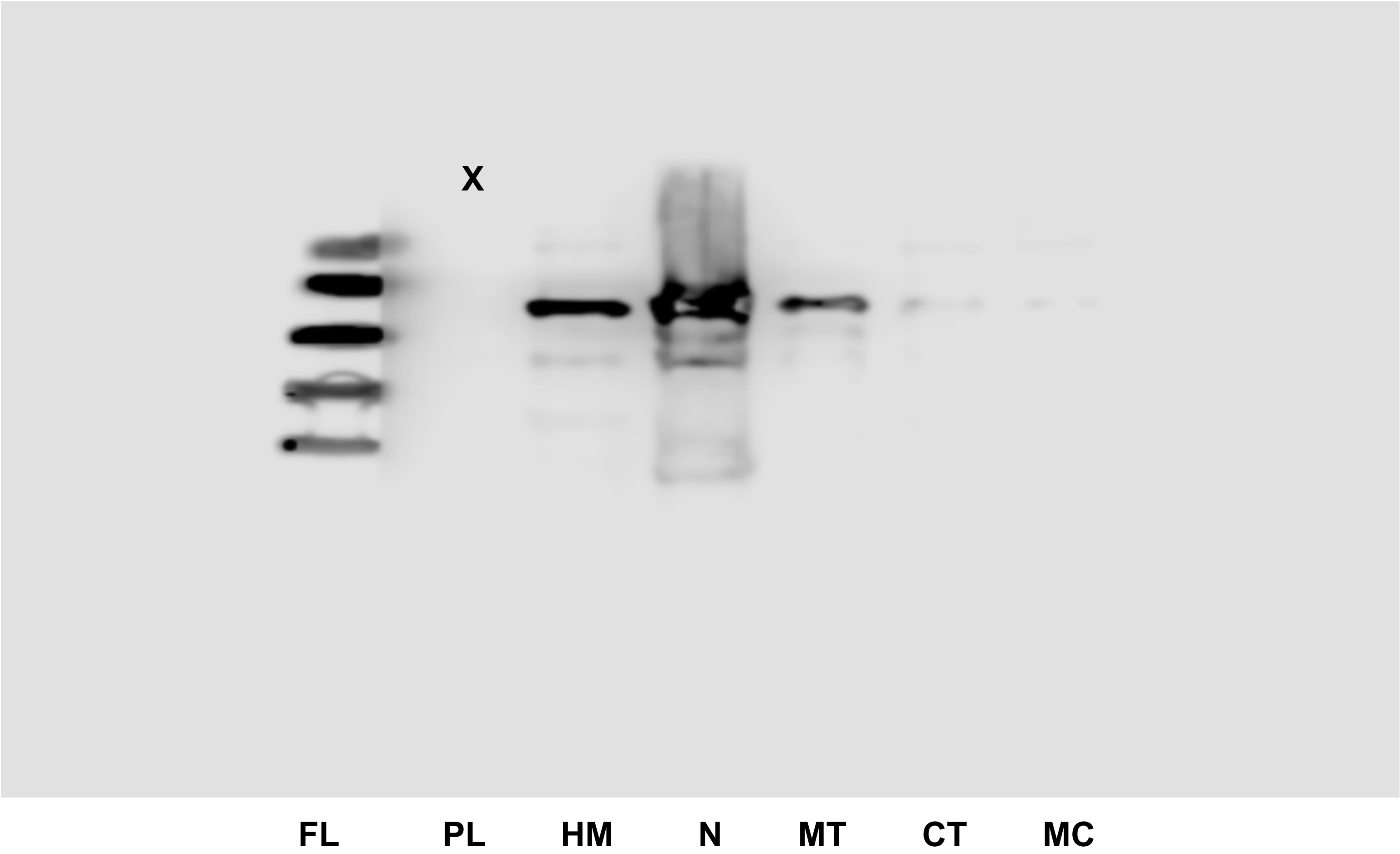

S3C Fig. Lanes: FL: plastic fluorescent marker, PL: protein ladder, HM: homogenate, N: Nuclear, MT: mitochondrial and MC: microsomal pellets, CT: cytosol.

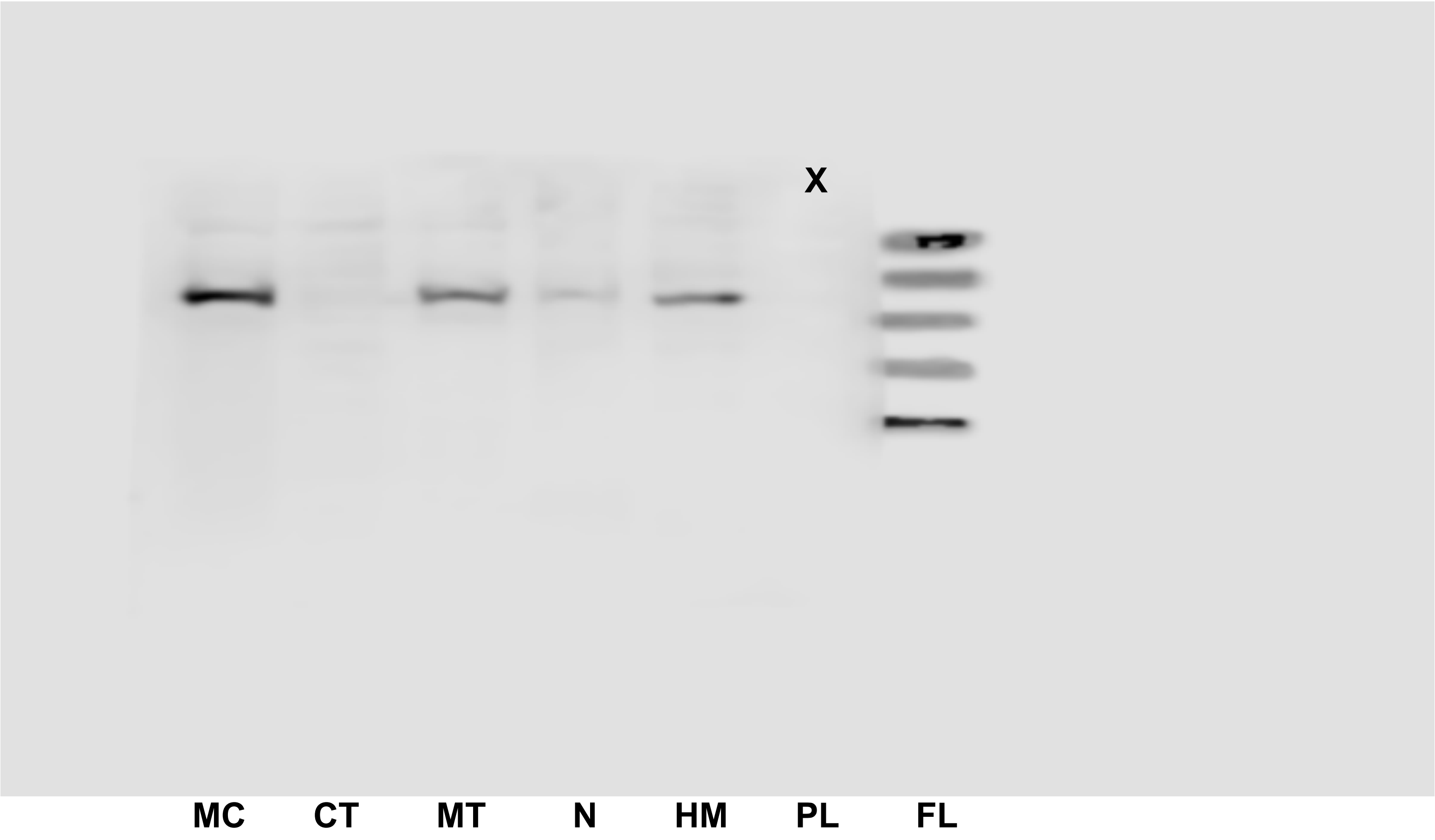

S3D Fig. Lanes: FL: plastic fluorescent marker, PL: protein ladder, HM: homogenate, N: Nuclear, MT: mitochondrial and MC: microsomal pellets, CT: cytosol.

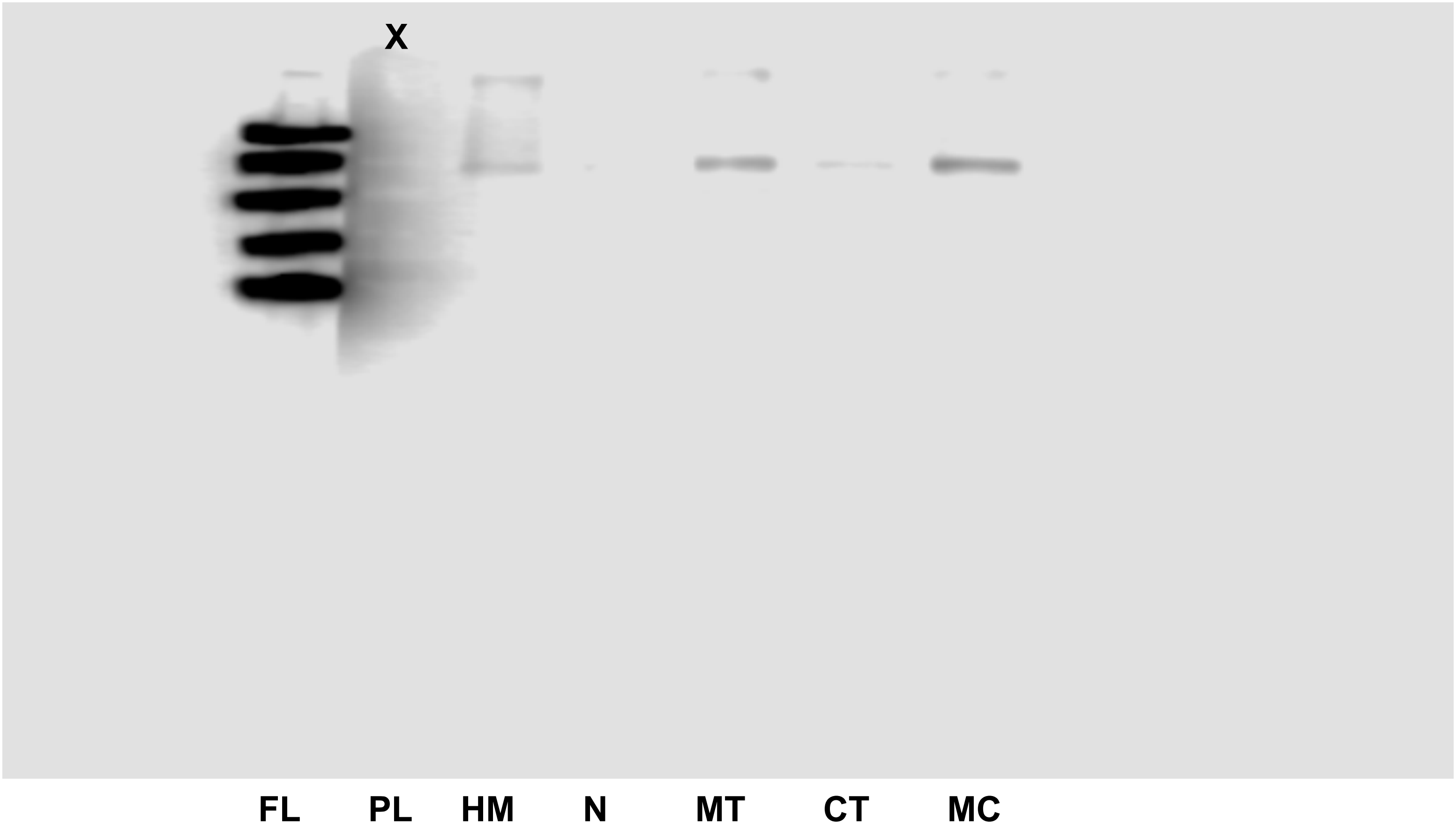

S3E Fig. Lanes: FL: plastic fluorescent marker, PL: protein ladder, HM: homogenate, N: Nuclear, MT: mitochondrial and MC: microsomal pellets, CT: cytosol.

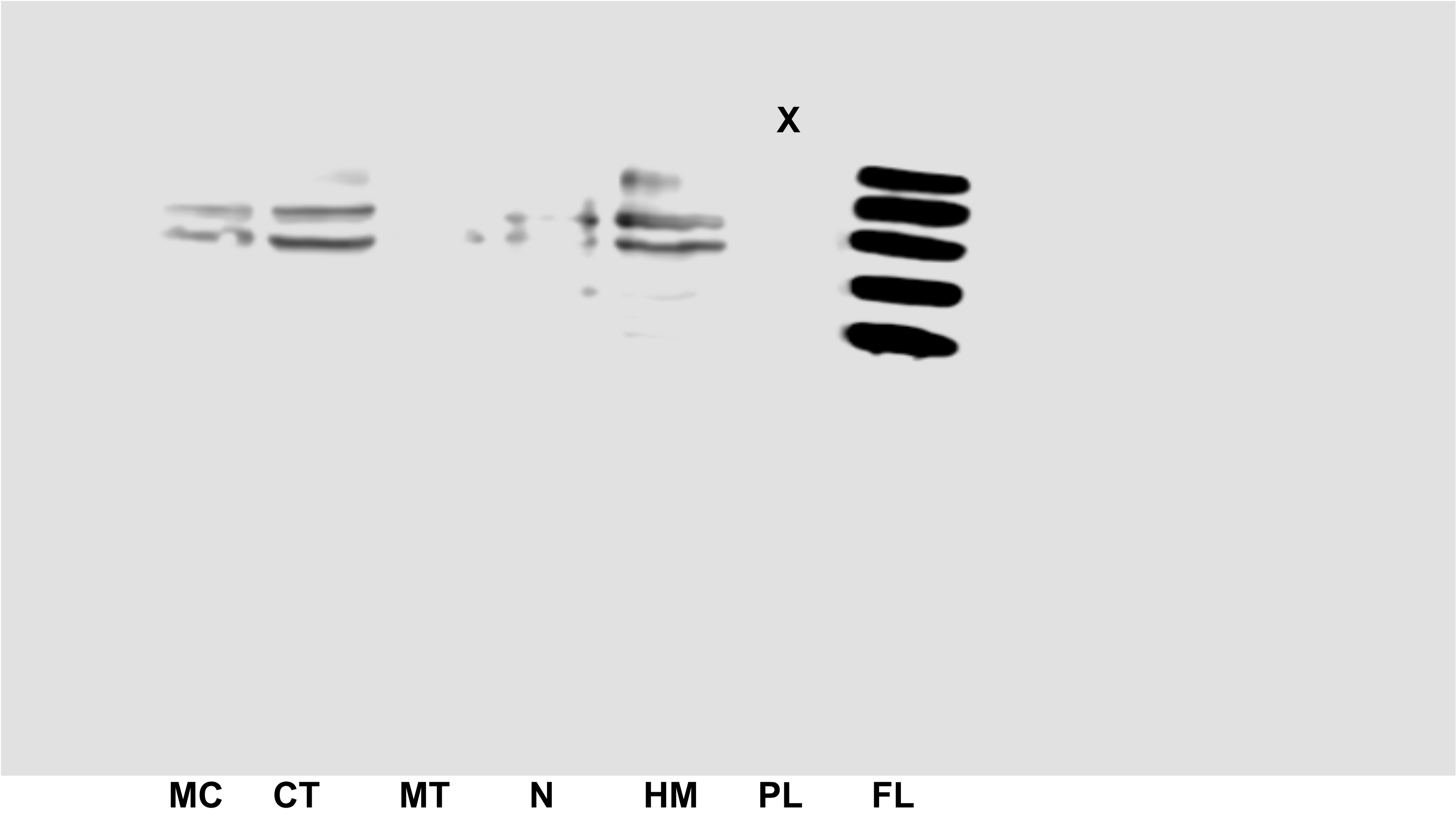

S4C Fig. Lanes: PL: protein ladder, 1-9: Fractions collected from 15-60% gradient during separation of the microsomal precipitate, and numbered from the top to the bottom of the tube.

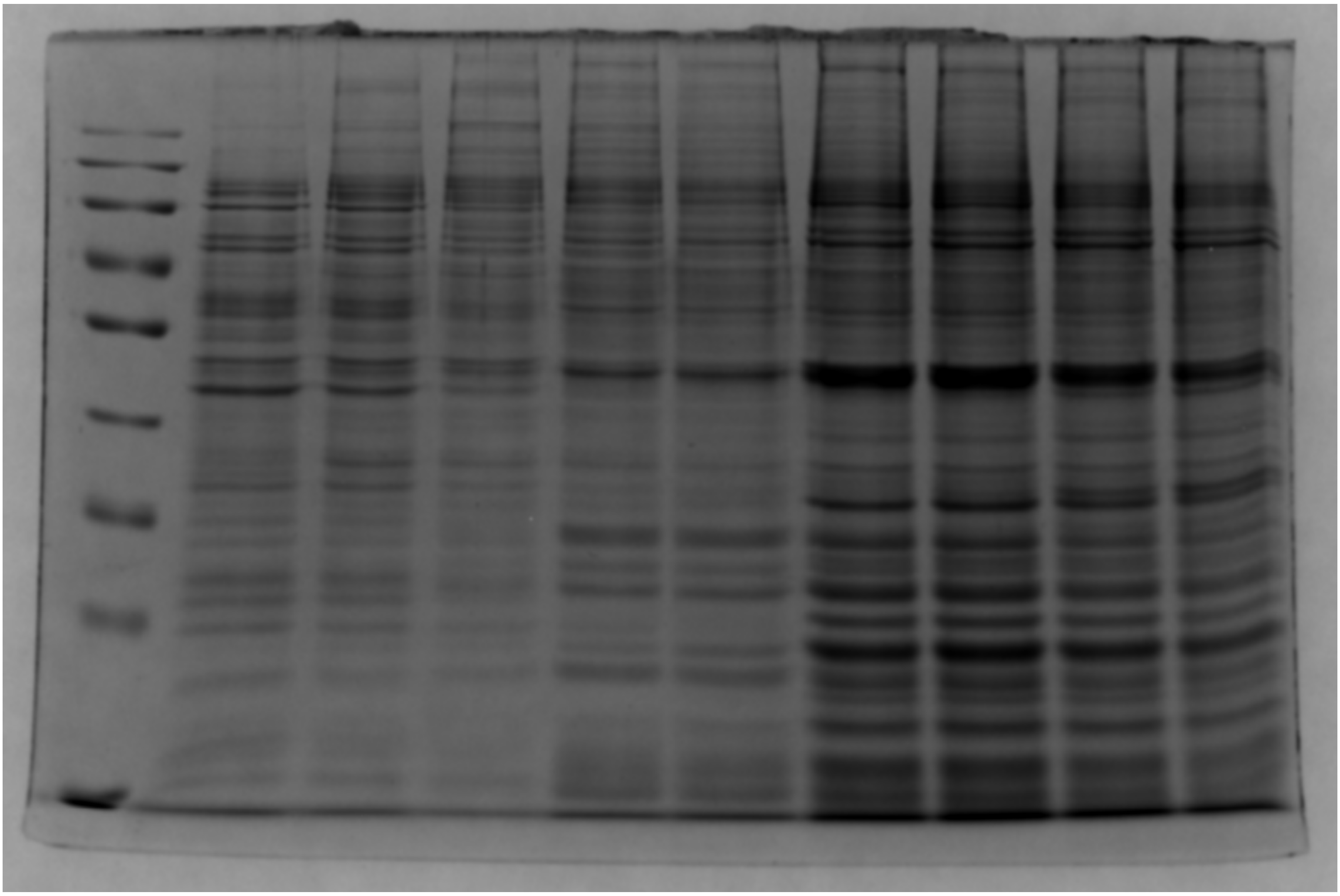

PL 1 2 3 4 5 6 7 8 9

S4D Fig. Lanes: PL: protein ladder, 10-18: Fractions collected from 15-60% sucrose gradient during separation of the microsomal precipitate, and numbered from the top to the bottom of the tube.

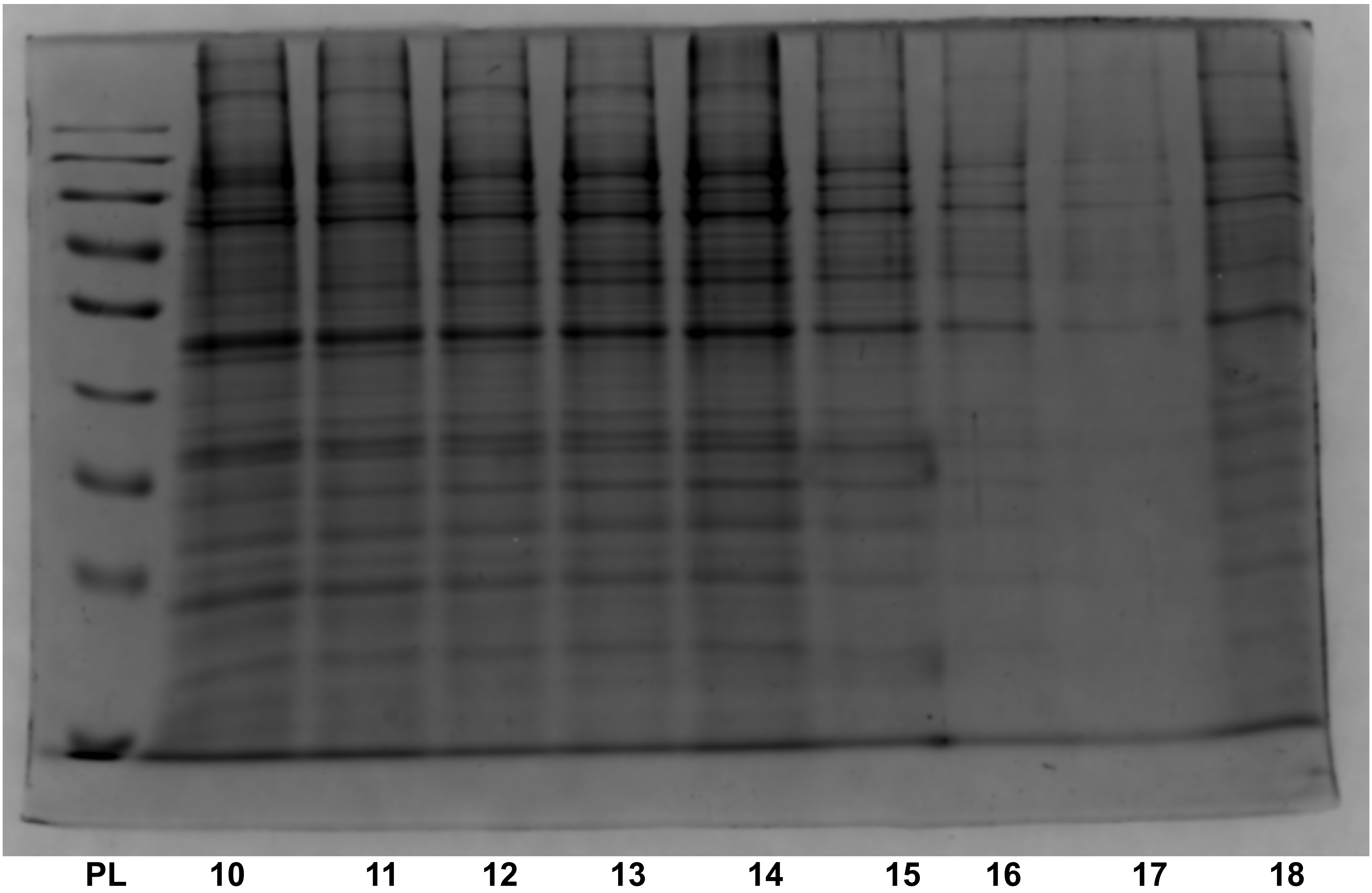

S5A Fig. Lanes: FL: plastic fluorescent marker, PL: protein ladder, 6-15: Fractions collected from a 15-60% gradient during separation of the microsomal pellet, and numbered from the top to the bottom of the tube .

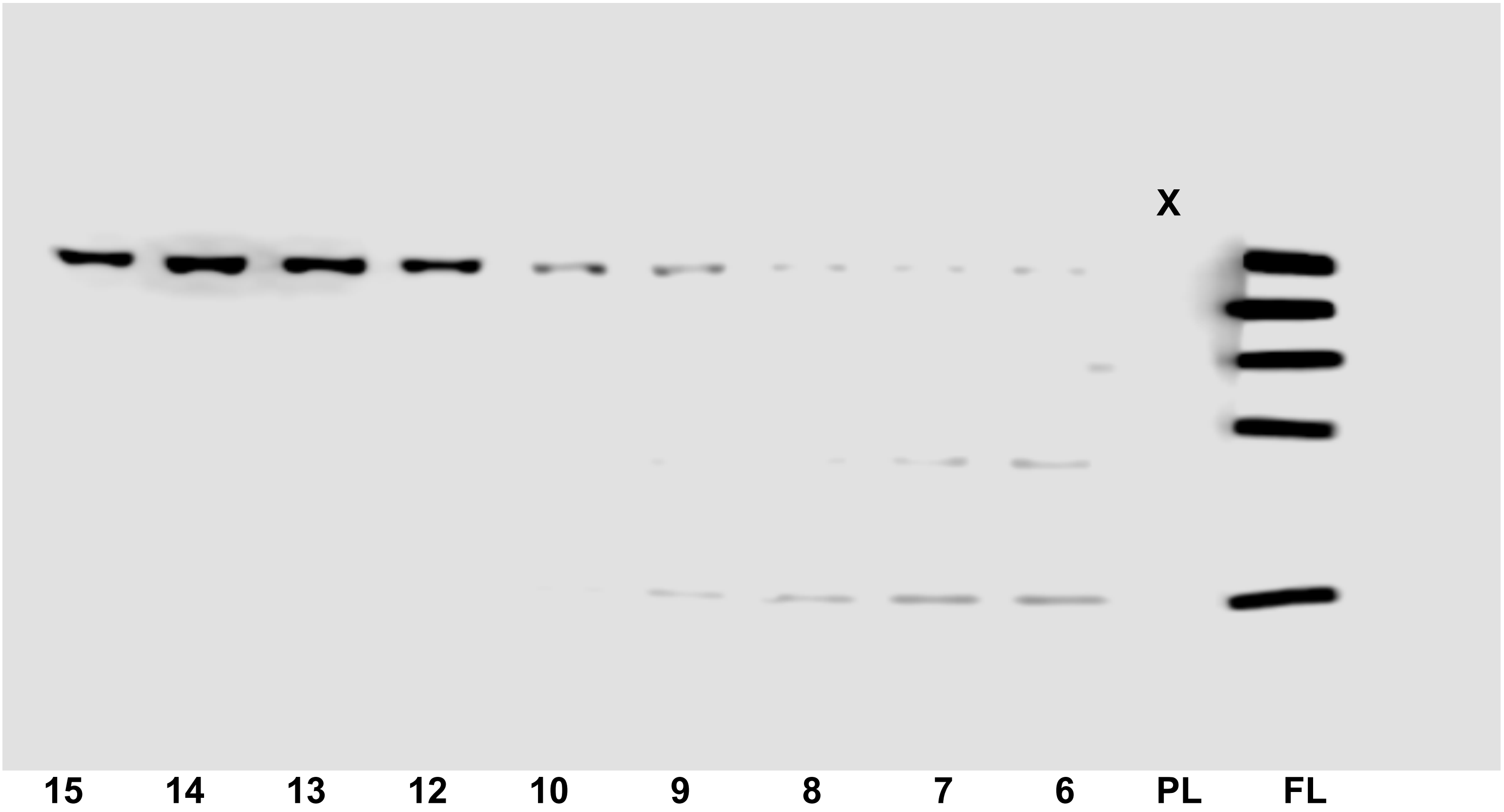

S5B Fig. Lanes: FL: plastic fluorescent marker, PL: protein ladder, HM: homogenate, 2-9: Fractions collected from a 15-60% gradient during separation of the microsomal pellet, and numbered from the top to the bottom of the tube.

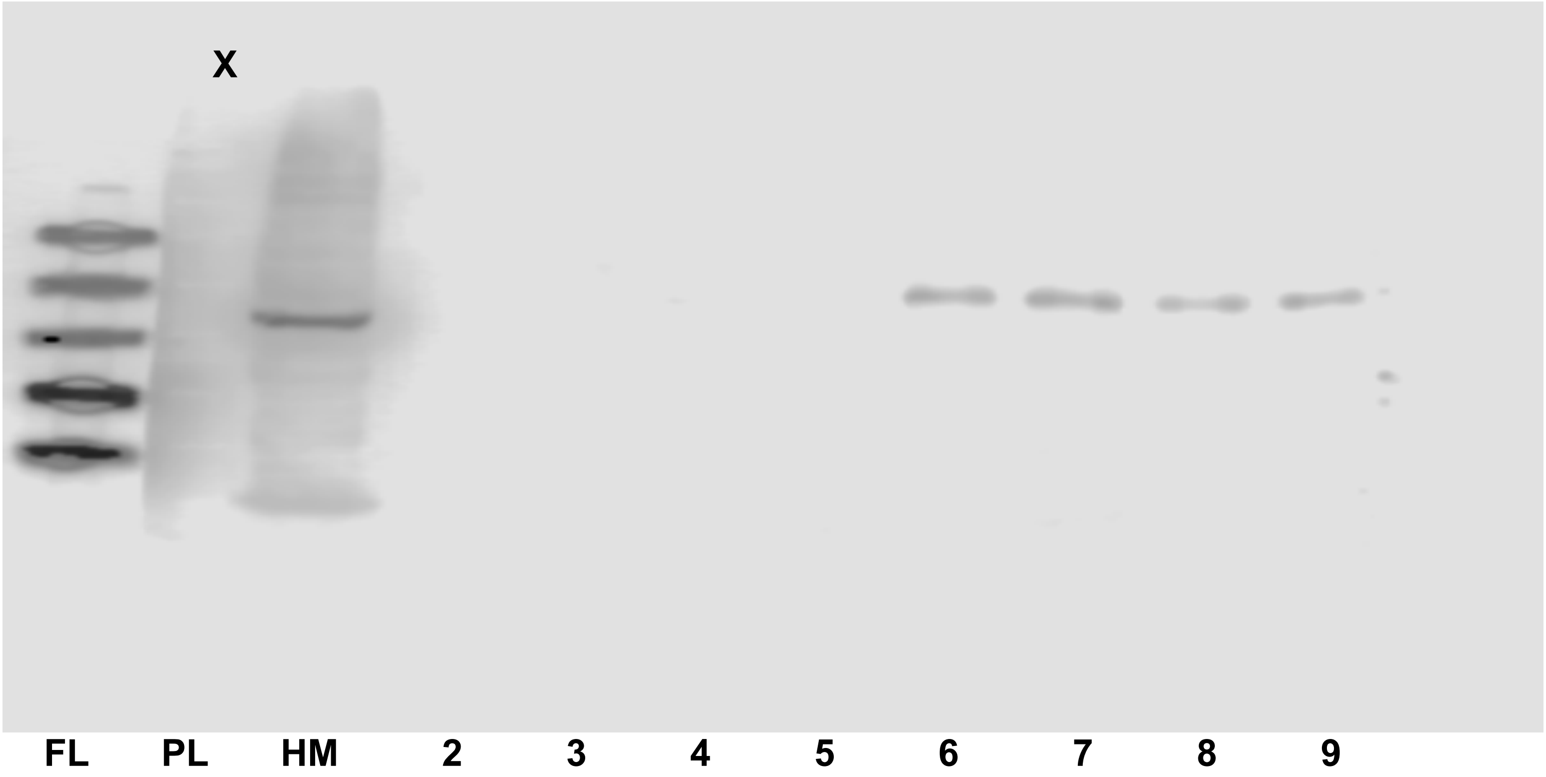

S5C Fig. Lanes: FL: plastic fluorescent marker, PL: protein ladder, HM: homogenate, 10-16: Fractions collected from a 15-60% gradient during separation of the microsomal pellet, and numbered from the top to the bottom of the tube.

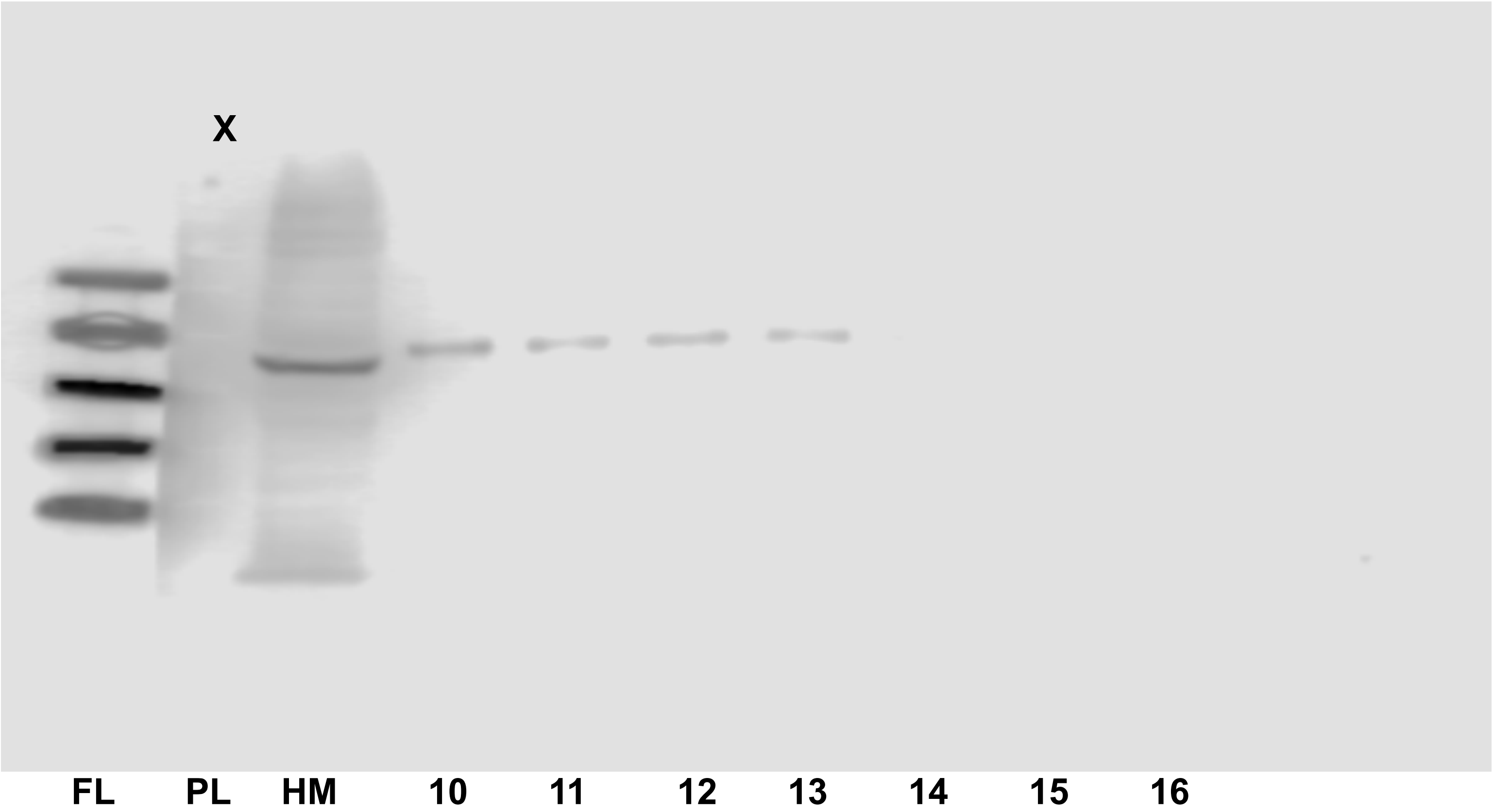

S5D Fig. Lanes: FL: plastic fluorescent marker, PL: protein ladder, HM: homogenate, 2-9: Fractions collected from a 15-60% gradient during separation of the microsomal pellet, and numbered from the top to the bottom of the tube.

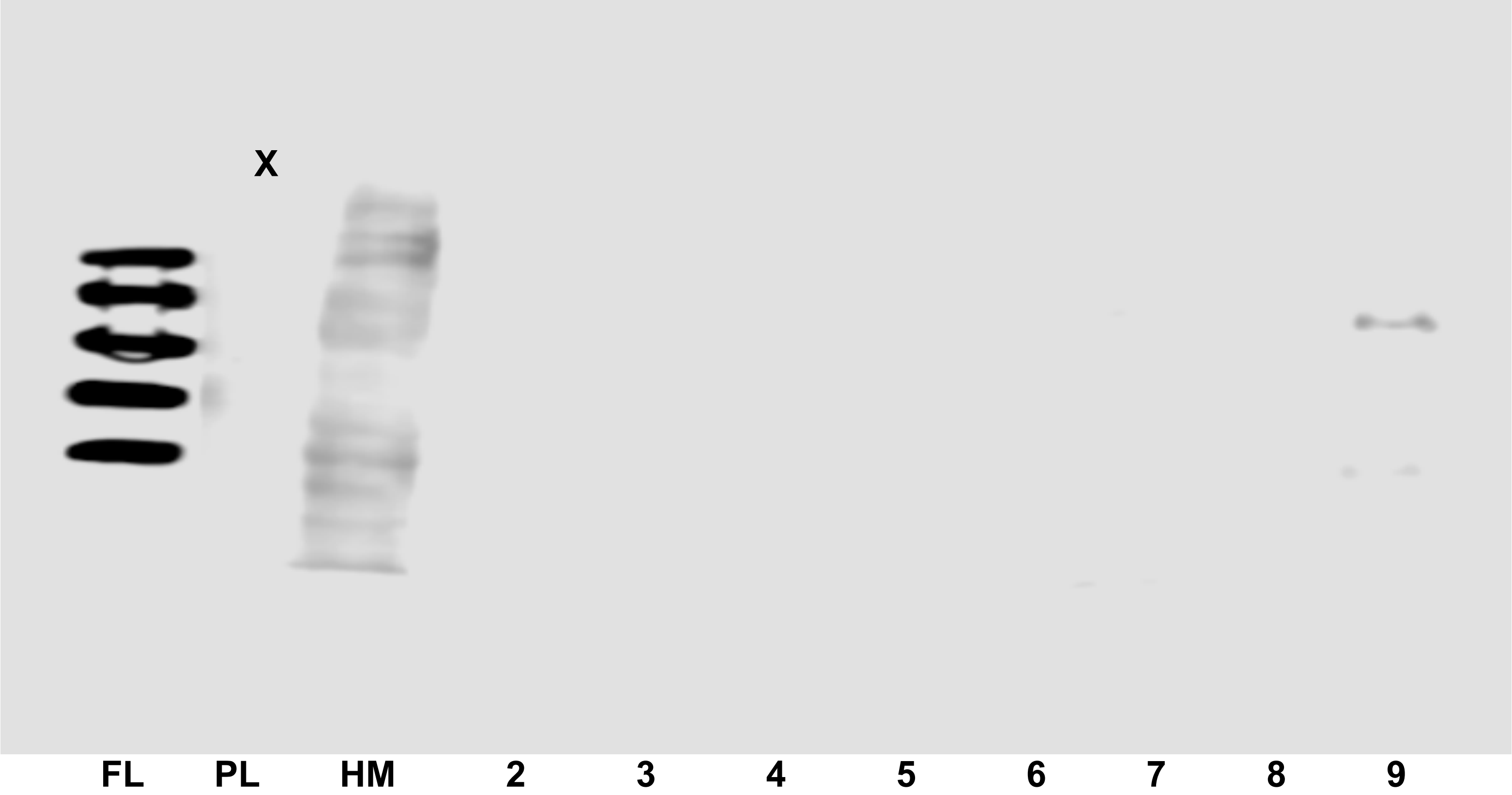

S5E Fig. Lanes: FL: plastic fluorescent marker, PL: protein ladder, HM: homogenate, 10-17: Fractions collected from a 15-60% gradient during separation of the microsomal pellet, and numbered from the top to the bottom of the tube.

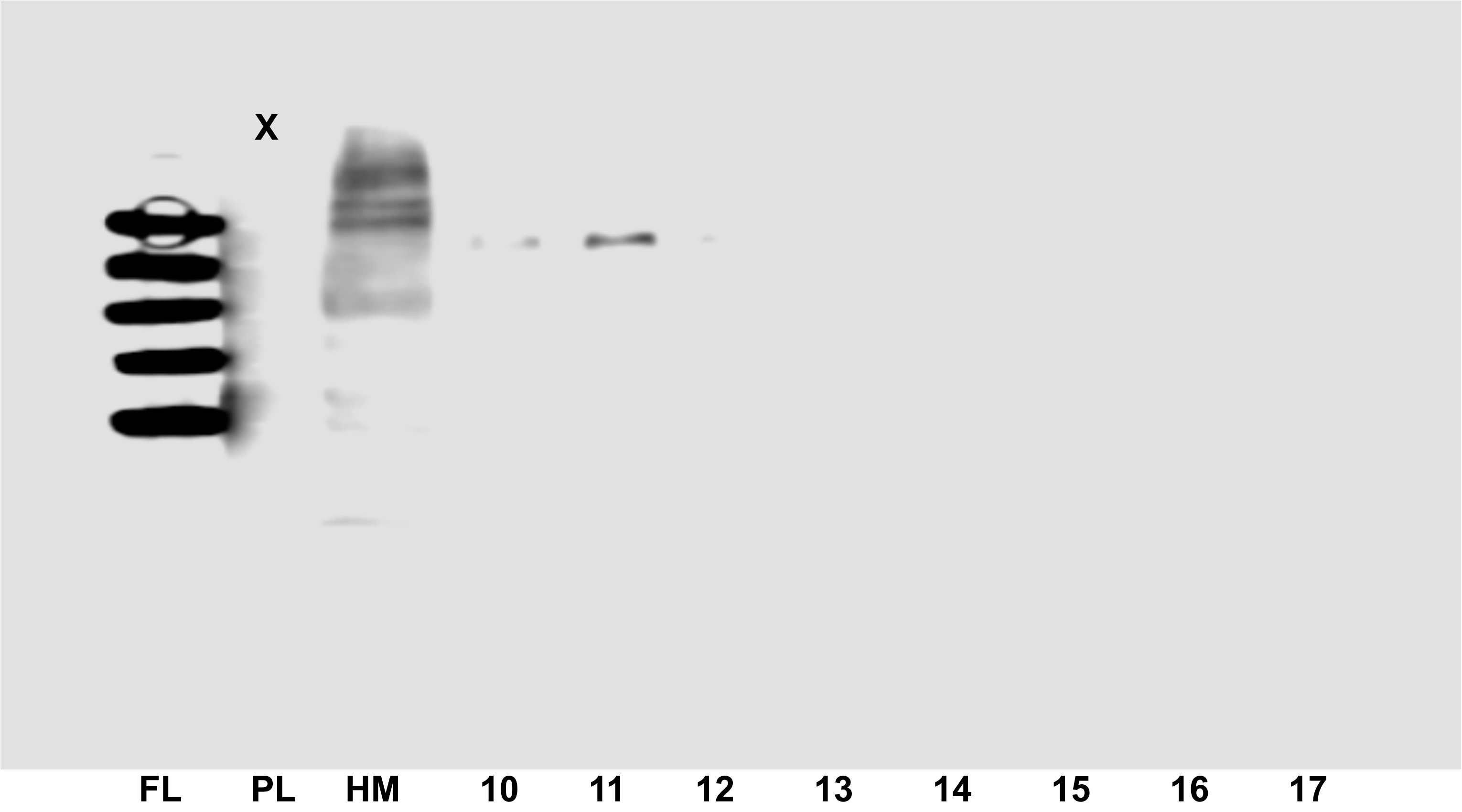

S5F Fig. Lanes: FL: plastic fluorescent marker, PL: protein ladder, HM: homogenate, 2-5: Fractions collected from a 15-60% gradient during separation of the microsomal pellet, and numbered from the top to the bottom of the tube.

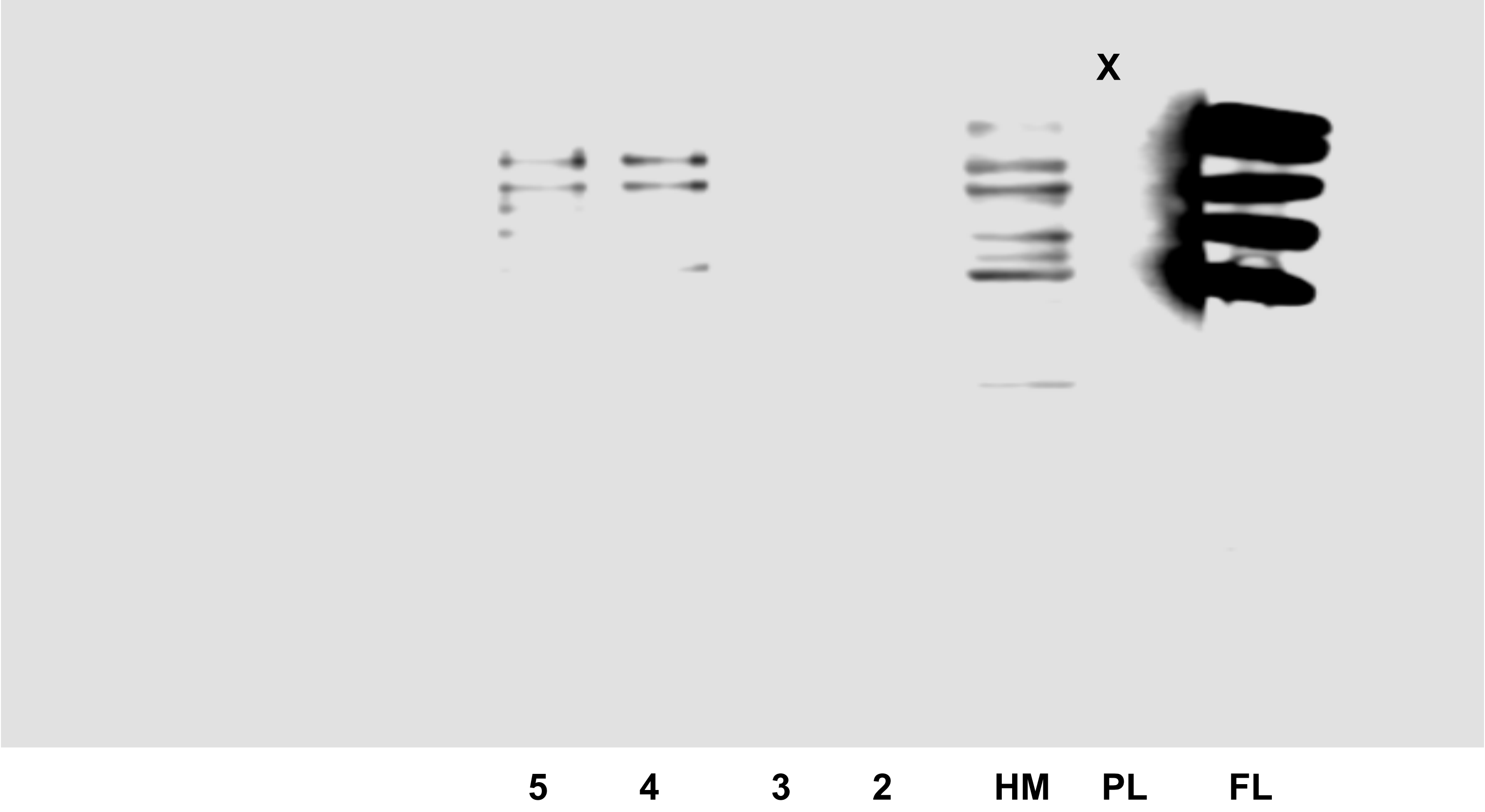

S5G Fig. Lanes: FL: plastic fluorescent marker, PL: protein ladder, 6-15: Fractions collected from a 15-60% gradient during separation of the microsomal pellet, and numbered from the top to the bottom of the tube.

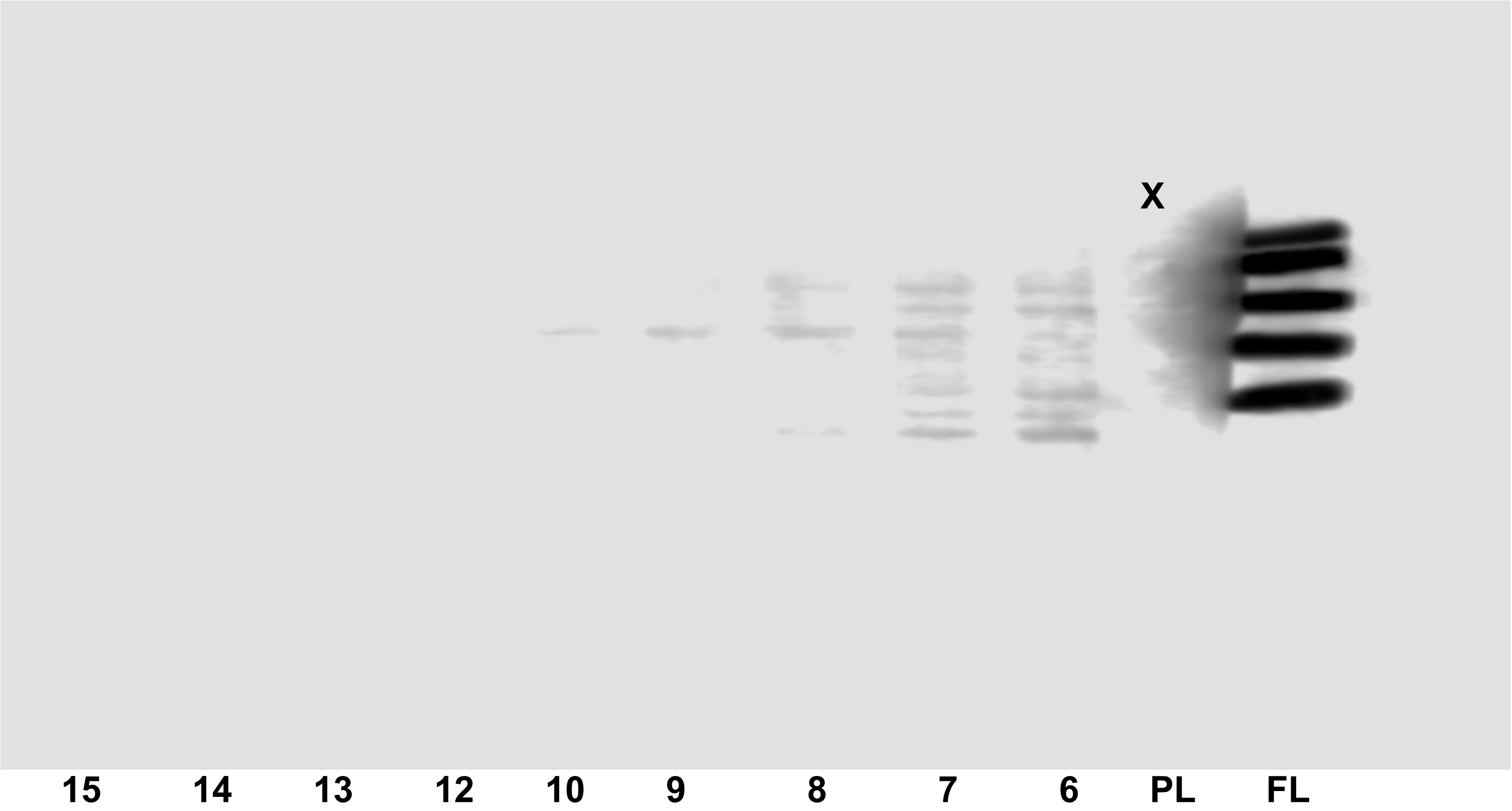

S6D Fig. Lanes: PL: protein ladder, P1-P9: Protein peaks collected from 30-60% y 10-60% gradients during separation of nuclear, mitochondrial and microsomal precipitates, and numbered from the top to the bottom of the tubes.

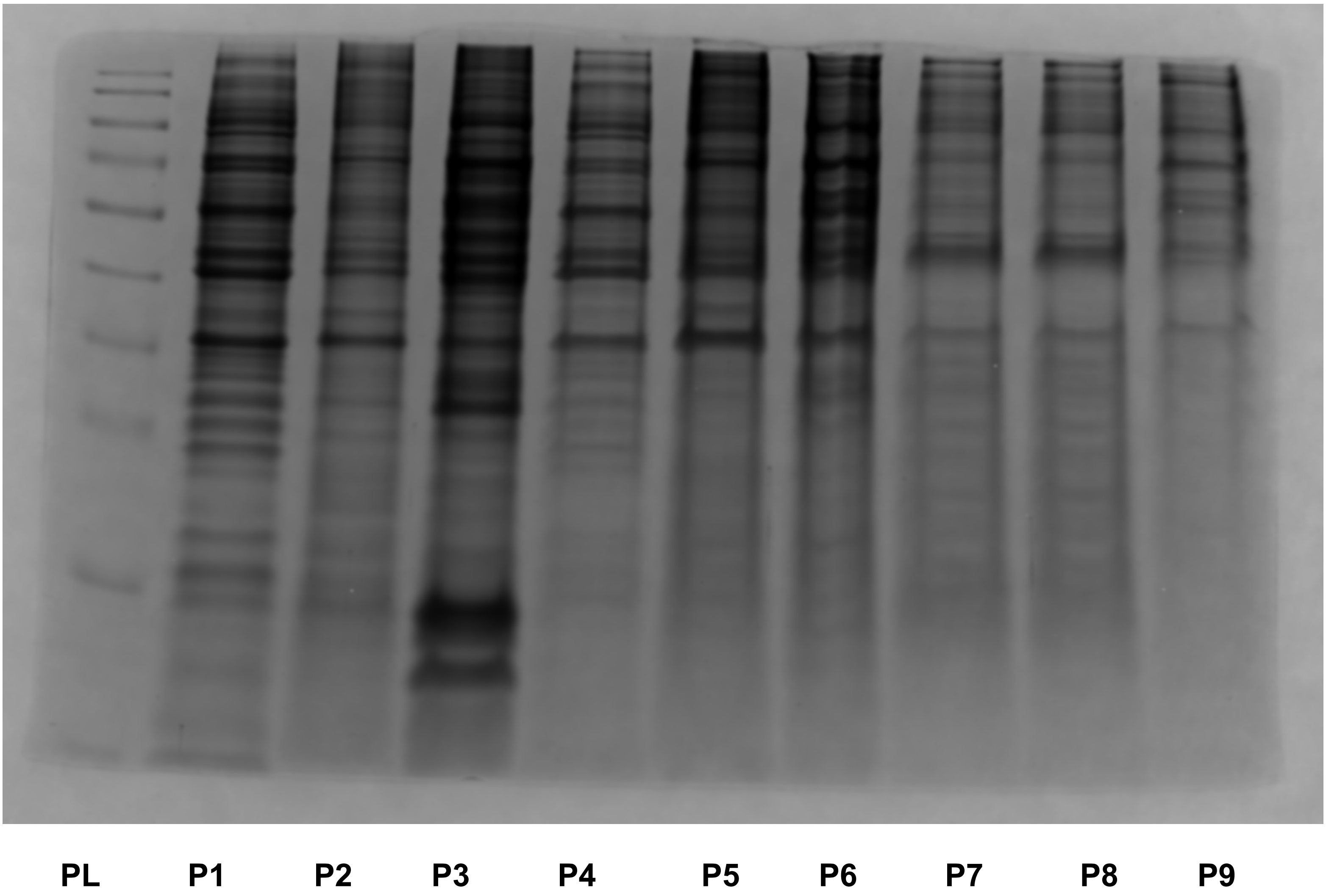

S7A Fig. Lanes: FL: plastic fluorescent marker, PL: protein ladder, P1-P9: protein peaks collected from 30-60% and 10-60% gradients during separation of nuclear, mitochondrial and microsomal pellets, and numbered from the top to the bottom of the tubes.

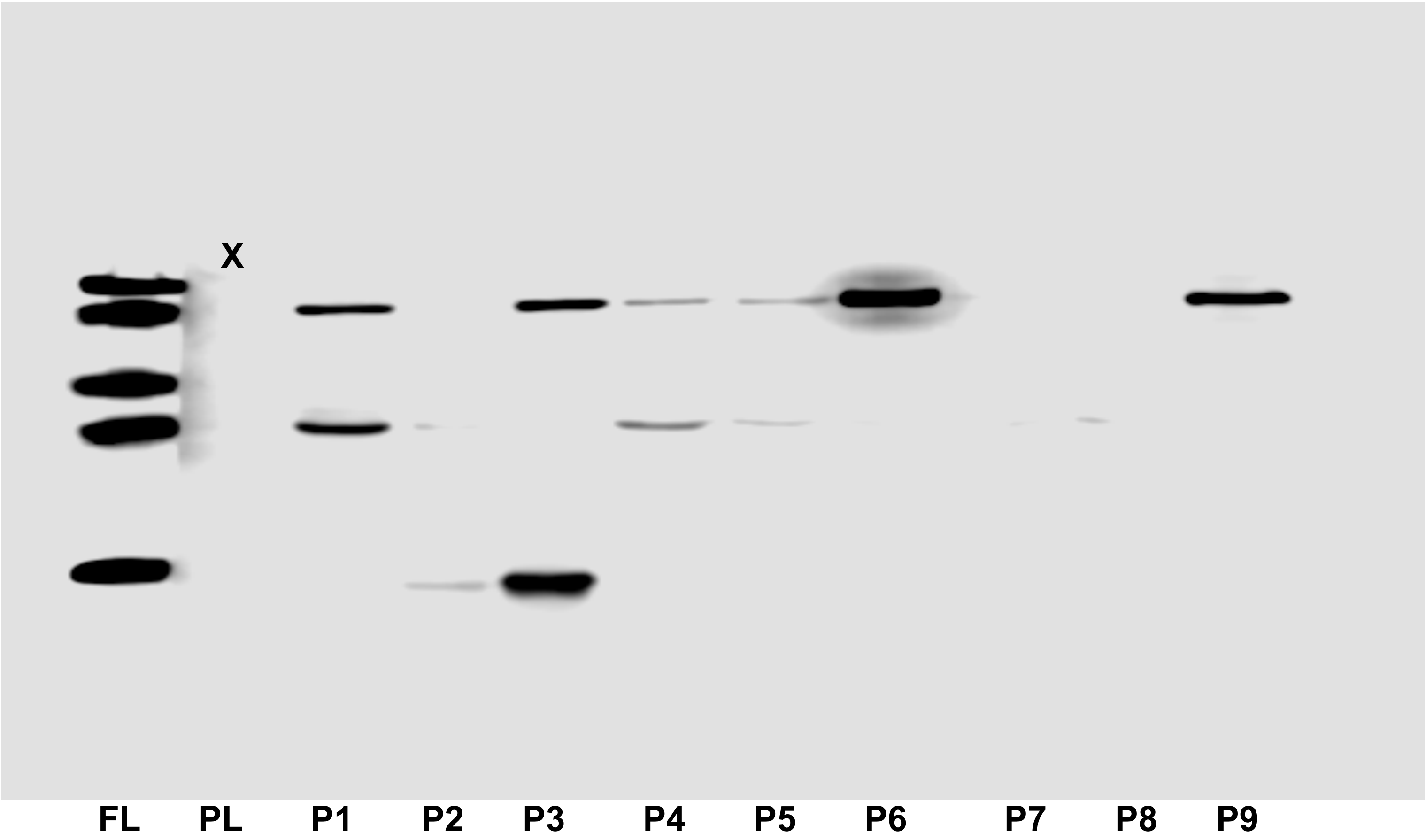

S7B Fig. Lanes: FL: plastic fluorescent marker, PL: protein ladder, P1-P6: protein peaks collected from 30-60% and 10-60% gradients during separation of nuclear, mitochondrial and microsomal pellets, and numbered from the top to the bottom of the tubes.

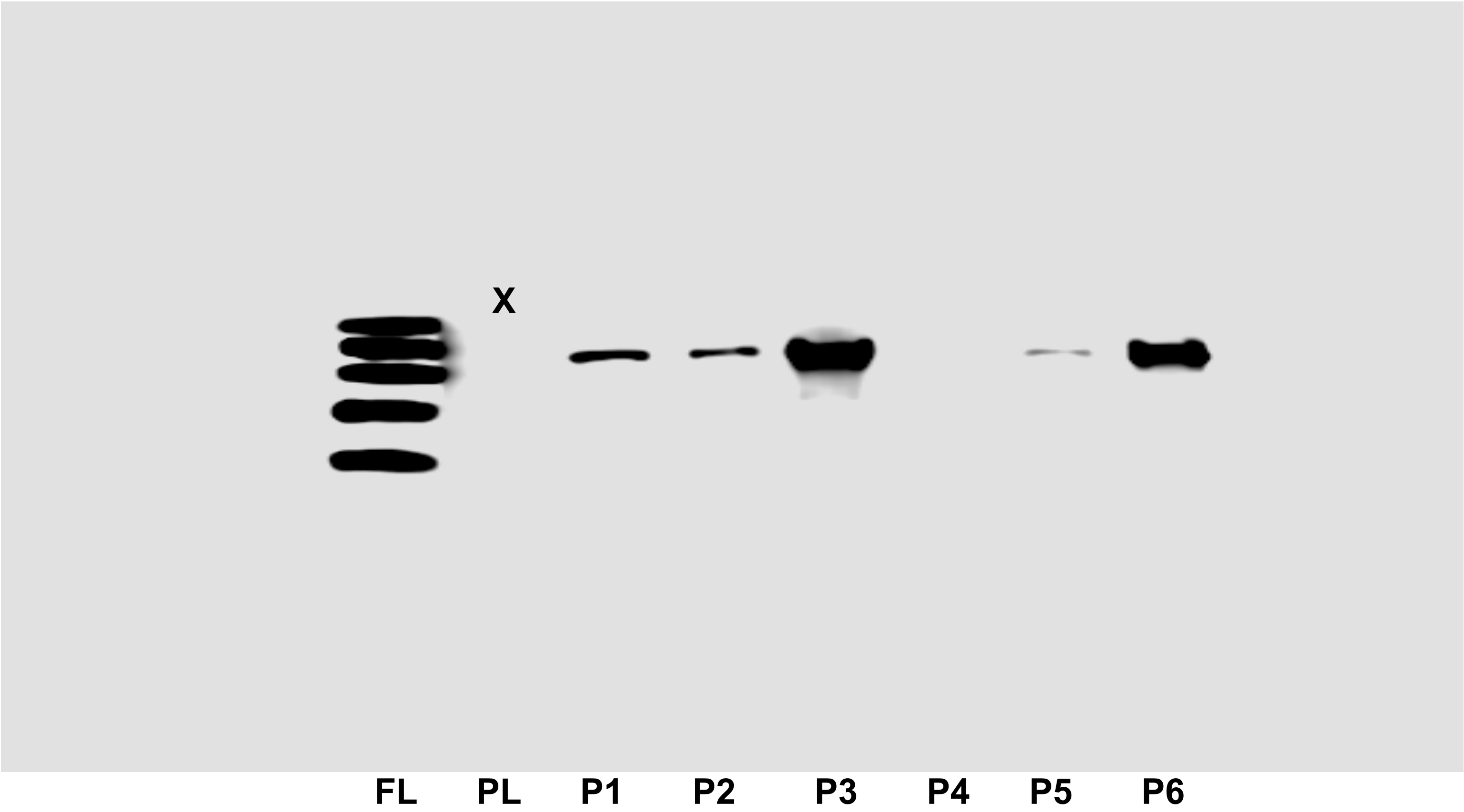

S7C Fig. Lanes: FL: plastic fluorescent marker, PL: protein ladder, P1-P9: protein peaks collected from 30-60% and 10-60% gradients during separation of nuclear, mitochondrial and microsomal pellets, and numbered from the top to the bottom of the tubes.

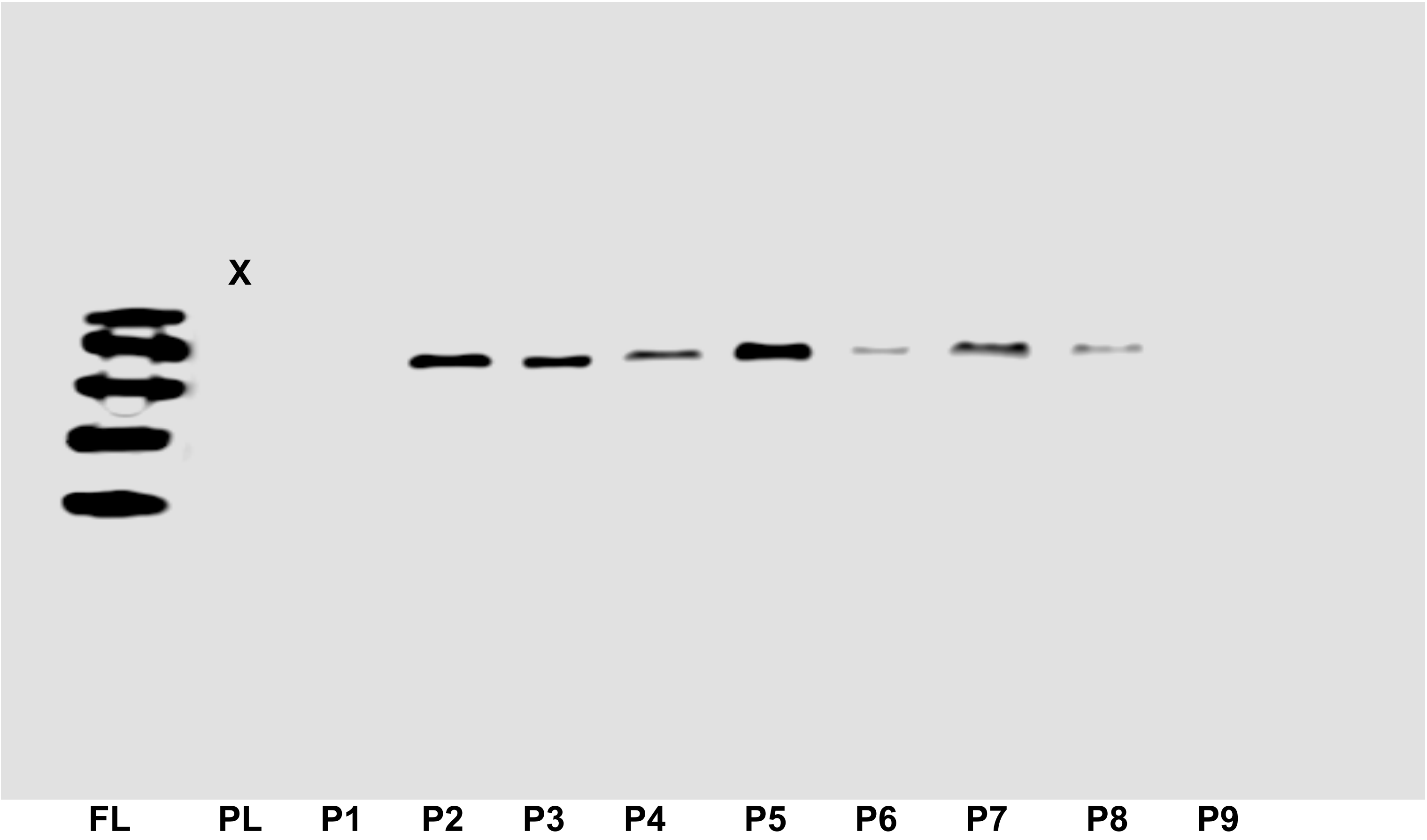

S7D Fig. Lanes: FL: plastic fluorescent marker, PL: protein ladder, P4-P9: protein peaks collected from 30-60% and 10-60% gradients during separation of nuclear, mitochondrial and microsomal pellets, and numbered from the top to the bottom of the tubes.

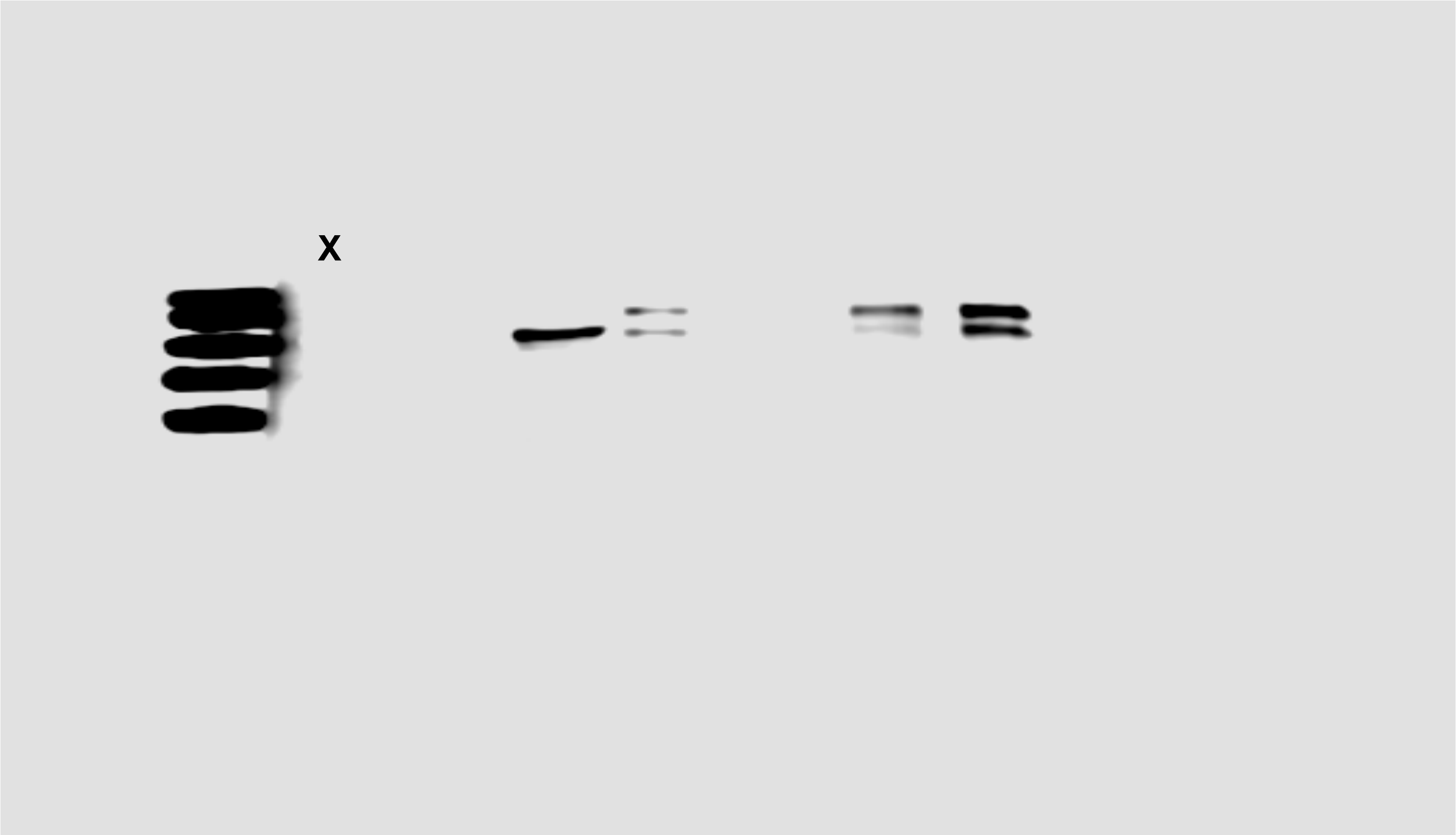

FL PL P4 P5 P6 P7 P8 P9

S7E Fig. Lanes: FL: plastic fluorescent marker, PL: protein ladder, P7-P9: protein peaks collected from 30-60% and 10-60% gradients during separation of nuclear, mitochondrial and microsomal pellets, and numbered from the top to the bottom of the tubes.

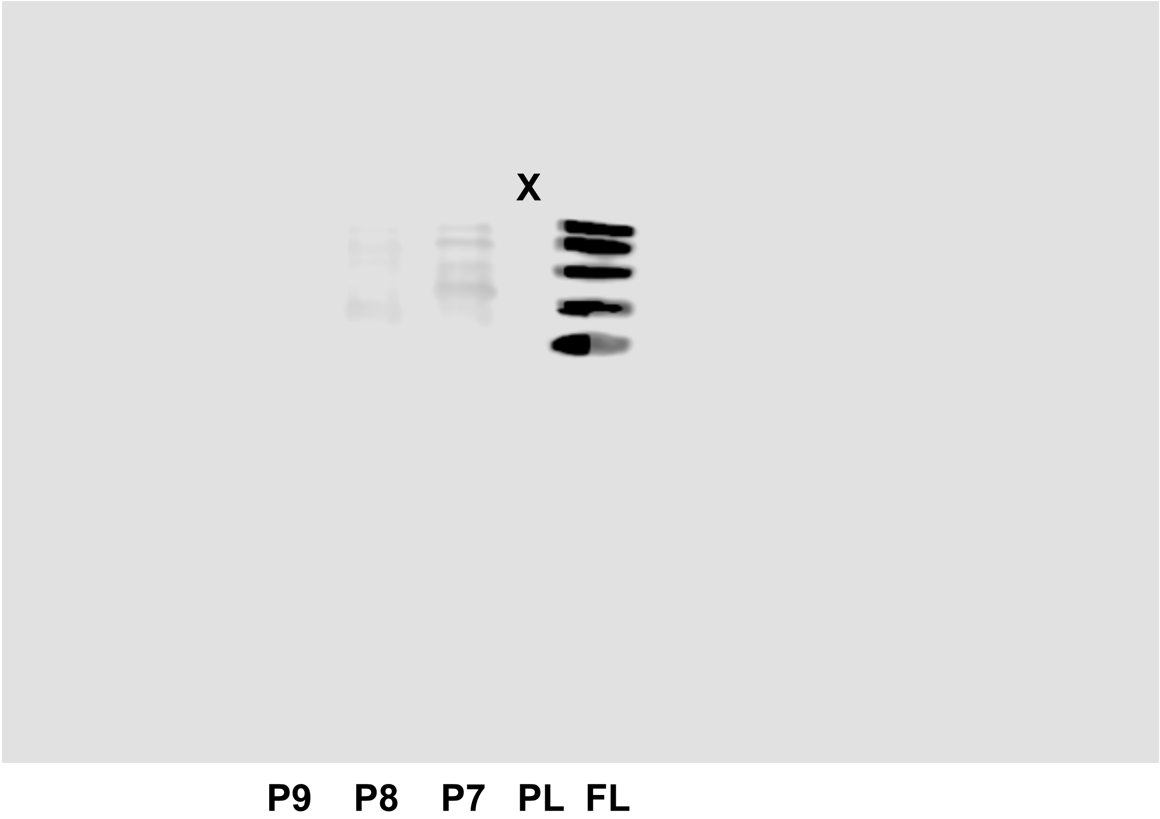

Supplement: S1 Raw images — Gels were stained with Coomassie Brilliant Blue G 250 from Sigma-Aldrich (B8647), and images acquired in a Gel DocTM EZ imager by using Image Lab software, v6.0.1 (Bio-Rad, CA, USA). (PDF) [file pone.0237930.s018.pdf]
